# Exendin-4 stimulates autophagy in pancreatic β-cells via the RAPGEF/EPAC-Ca^2+^-PPP3/calcineurin-TFEB axis

**DOI:** 10.1080/15548627.2021.1956123

**Published:** 2021-08-02

**Authors:** Francesco P Zummo, Stanislaus I Krishnanda, Merilin Georgiou, Finbarr PM O’Harte, Vadivel Parthsarathy, Kirsty S Cullen, Minna Honkanen-Scott, James AM Shaw, Penny E Lovat, Catherine Arden

**Affiliations:** a Biosciences Institute, Newcastle University, Newcastle Upon Tyne, UK; b Department of Medicine, Universitas Indonesia, Jakarta, Indonesia; c The SAAD Centre for Pharmacy & Diabetes, Ulster University, Coleraine, Northern Ireland, UK; d Translational and Clinical Research Institute, Newcastle University, Newcastle Upon Tyne, UK

**Keywords:** Autophagy, diabetes, GLP1R agonists, pancreatic β-cell, PPP3/calcineurin, RAPGEF4, TFEB

## Abstract

Macroautophagy/autophagy is critical for the regulation of pancreatic β-cell mass and its deregulation has been implicated in the pathogenesis of type 2 diabetes (T2D). We have previously shown that treatment of pancreatic β-cells with the GLP1R (glucagon like peptide 1 receptor) agonist exendin-4 stimulates autophagic flux in a setting of chronic nutrient excess. The aim of this study was to identify the underlying pathways contributing to enhanced autophagic flux.

Pancreatic β-cells (INS-1E),mouse and human islets were treated with glucolipotoxic stress (0.5 mM palmitate and 25 mM glucose) in the presence of exendin-4. Consistent with our previous work, exendin-4 stimulated autophagic flux. Using chemical inhibitors and siRNA knockdown, we identified RAPGEF4/EPAC2 (Rap guanine nucleotide exchange factor 4) and downstream calcium signaling to be essential for regulation of autophagic flux by exendin-4. This pathway was independent of AMPK and MTOR signaling. Further analysis identified PPP3/calcineurin and its downstream regulator TFEB (transcription factor EB) as key proteins mediating exendin-4 induced autophagy. Importantly, inhibition of this pathway prevented exendin-4-mediated cell survival and overexpression of TFEB mimicked the cell protective effects of exendin-4 in INS-1E and human islets. Moreover, treatment of *db/db* mice with exendin-4 for 21 days increased the expression of lysosomal markers within the pancreatic islets. Collectively our data identify the RAPGEF4/EPAC2-calcium-PPP3/calcineurin-TFEB axis as a key mediator of autophagic flux, lysosomal function and cell survival in pancreatic β-cells. Pharmacological modulation of this axis may offer a novel therapeutic target for the treatment of T2D.

**Abbreviations**: AKT1/protein kinase B: AKT serine/threonine kinase 1; AMPK: 5’ AMP-activated protein kinase; CAMKK: calcium/calmodulin-dependent protein kinase kinase; cAMP: cyclic adenosine monophosphate; CASP3: caspase 3; CREB: cAMP response element-binding protein; CTSD: cathepsin D; Ex4: exendin-4(1-39); GLP-1: glucagon like peptide 1; GLP1R: glucagon like peptide 1 receptor; GLT: glucolipotoxicity; INS: insulin; MTOR: mechanistic target of rapamycin kinase; NFAT: nuclear factor of activated T-cells; PPP3/calcineurin: protein phosphatase 3; PRKA/PKA: protein kinase cAMP activated; RAPGEF3/EPAC1: Rap guanine nucleotide exchange factor 3; RAPGEF4/EPAC2: Rap guanine nucleotide exchange factor 4; SQSTM1/p62: sequestosome 1; T2D: type 2 diabetes; TFEB: transcription factor EB

## Introduction

Macroautophagy/autophagy is a highly conserved pathway that serves to recycle unwanted or damaged organelles to provide energy to maintain intracellular homeostasis and sustain core metabolic functions [1,2]. Fusion of the lysosomal and autophagosomal compartments is a critical step in the process and is highly dependent on lysosomal function [3]. Although classically considered a pro-survival mechanism active during nutrient deprivation, autophagy can also serve to remove superfluous nutrients, damaged/dysfunctional organelles or misfolded proteins, particularly in metabolically active tissues [4]. Accordingly, a role for autophagy in regulating cell survival in response to increased circulating nutrients has been proposed [5].

Pancreatic β-cells play a critical role in blood glucose homeostasis through the release of INS (insulin) in response to elevated glucose concentrations [6]. Failure of glucose to invoke an appropriate INS secretory response is central to the development of type 2 diabetes (T2D) [7] through a progressive decline of β-cell function, exhaustion and eventual β-cell demise [7,8]. Increased circulating nutrients particularly lipid, due to obesity and/or high fat diet, are major drivers in this process [9]. Due to high rates of protein synthesis, β-cells require robust degradation mechanisms for the disposal of misfolded/denatured proteins [10]. Autophagy appears critical for basal β-cell physiology and survival because β-cell specific ablation of the key autophagic protein ATG7 caused accumulation of misfolded proteins/damaged organelles which was associated with decreased INS secretion and decreased β-cell mass [11,12]. Moreover, β-cell autophagy is also stimulated by excess nutrients [12–15] and studies using β-cell specific *atg7* knockout mice show that autophagy is essential for the compensatory increase in β-cell mass in response to high-fat diet [12]. However, other studies [16,17] including our own [18], have shown that upon sustained nutrient excess, autophagic flux becomes impaired. This is consistent with the accumulation of autophagosomal structures in islets from T2D patients [18–20]. Continued stimulation of this deregulated pathway eventually leads to accumulation of defective lysosomes, which can induce a pro-death stimulus [18]. In this setting, it is proposed that autophagy is detrimental and therefore contributes to loss of functional β-cell mass [16–20].

Pharmacological approaches to restore autophagic flux have been shown to improve β-cell function/survival. Studies using rapamycin or carbamazepine to stimulate autophagy prevented palmitate-induced β-cell death [21,22]. However, global targeting of this ubiquitous pathway is not recommended particularly since upregulation of autophagy is integral to the metastasis of many human cancers [23]. A more selective approach to target these pathways would be preferred. Recent research has identified GLP1R (glucagon like peptide 1 receptor) agonists, which are currently in clinical use for the treatment of T2D, as modulators of autophagy in several different tissues [24], including β-cells [18,25,26]. Our recent study showed that treatment with the GLP1R agonist exendin-4 (Ex4) restored the impairment in autophagic flux induced by nutrient excess through an improvement in lysosomal function and also stimulation of the autophagic pathway [18]. However, at present, the underlying pathways remain unidentified. The aim of the current study was to elucidate the pathways underlying the improvement in autophagic flux exerted by Ex4 in our β-cell model of nutrient excess.

## Results

### Signaling via RAPGEF/EPAC is essential for exendin-4 mediated autophagy and *β*-cell survival

**Figure 1.**
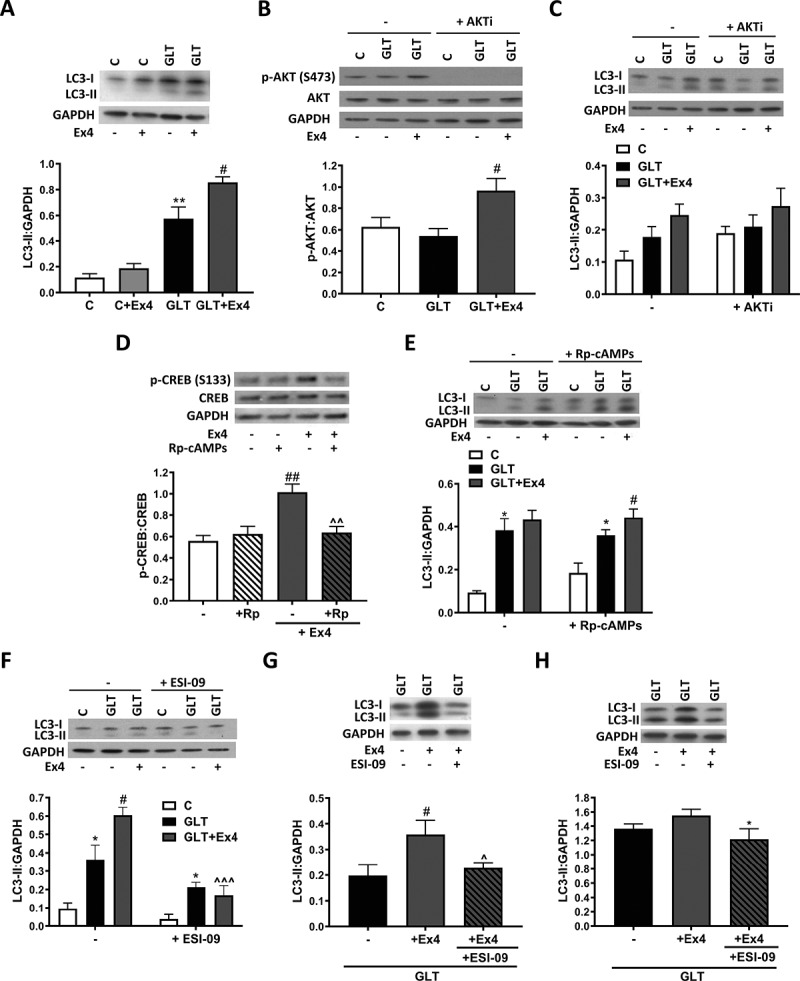
Exendin-4 stimulates autophagic flux via a PRKA-independent mechanism. (**A**) INS-1E treated with glucolipotoxicity (GLT, 25 mM glucose and 0.5 mM palmitate) without or with 100 nM Ex4 for 6 h. LC3 was analyzed by western blotting, quantified as LC3-II:GAPDH ratio. n = 6. (B and C) INS-1E were pre-incubated with 10 nM AKT inhibitor VIII (AKTi) for 1 h prior to the addition of glucolipotoxicity (GLT) without or with 100 nM Ex4 for 6 h. Changes in protein expression or phosphorylation were analyzed by western blotting, quantified relative to total AKT (B) or GAPDH (C). n = 5 (B) or 4 (C). (D) INS-1E were pre-incubated with 50 μM Rp-cAMPs for 1 h prior to the addition of 100 nM Ex4 for 30 min. p-CREB and total CREB were analyzed by western blotting and quantified as p-CREB/CREB ratio. n = 4. (E) INS-1E were pre-incubated with 50 μM Rp-cAMPs for 1 h prior to the addition of GLT -/+ Ex4 for 6 h. LC3 was analyzed by western blotting and quantified as LC3-II:GAPDH ratio. n = 6. (F-H) INS-1E (F), mouse (G), or human (H) islets were pre-incubated with 10 μM ESI-09 for 1 h prior to the addition of GLT -/+ Ex4 for either 6 (F) or 48 h (G and H). LC3 was analyzed by western blotting and quantified as LC3-II:GAPDH ratio. n = 4 (F and H) or 6 (G). Results are expressed as raw densitometry units corrected for the loading control as stated. All data are mean ± SEM of four to six individual experiments. Statistical analysis was performed using one-way (**A**, B, G, H) or two-way ANOVA (C, D, E, F) followed by Bonferroni’s post-hoc test. * P < 0.05, **P < 0.01 effect of GLT vs control; #P < 0.05, ##P < 0.01; ^ P < 0.05, ^^P < 0.01, ^^^P < 0.005 effect of inhibitor.

We first confirmed our prior findings [18] that in our β-cell model of nutrient excess (0.5 mM palmitate and 25 mM glucose, glucolipotoxicity [GLT]), Ex4 increased the conversion of LC3-I to LC3-II, a marker of increased autophagy (Figure 1A). We next explored the signaling pathways involved in this regulation. GLP1R agonists are known to exert their anti-diabetic actions via the activation of numerous signaling pathways, with the PtdIns3K-AKT-MTOR axis being critical for their anti-apoptotic actions [27]. In our model, Ex4 increased the phosphorylation of AKT at Ser473 (Figure 1B). Co-treatment with specific AKT inhibitor (Figure 1B) did not prevent the Ex4-mediated increase in LC3-II (Figure 1C) suggesting that the impact of Ex4 on autophagy is AKT-independent. We next explored a role for the cAMP pathway, which through its downstream targets PRKA/PKA (protein kinase cAMP activated) and RAPGEF4/EPAC2 (Rap guanine nucleotide exchange factor 4) has been shown to be essential for the impact of GLP1R agonists on both β-cell function and survival [28–30]. In our model of nutrient excess, Ex4 increased cAMP production (Fig. S1A). Inhibition of PRKA using Rp-cAMPs, as confirmed by the decrease in phosphorylation of the PRKA target CREB (cAMP responsive element binding protein) (Figure 1D) failed to inhibit the Ex4-mediated increase in LC3-II (Figure 1E). In contrast, inhibition of the RAPGEF pathway using the RAPGEF3-RAPGEF4 inhibitor ESI-09 prevented the Ex4-mediated increase in LC3-II in INS-1E (Figure 1F), mouse (Figure 1G) and human islets (Figure 1H).

**Figure 2.**
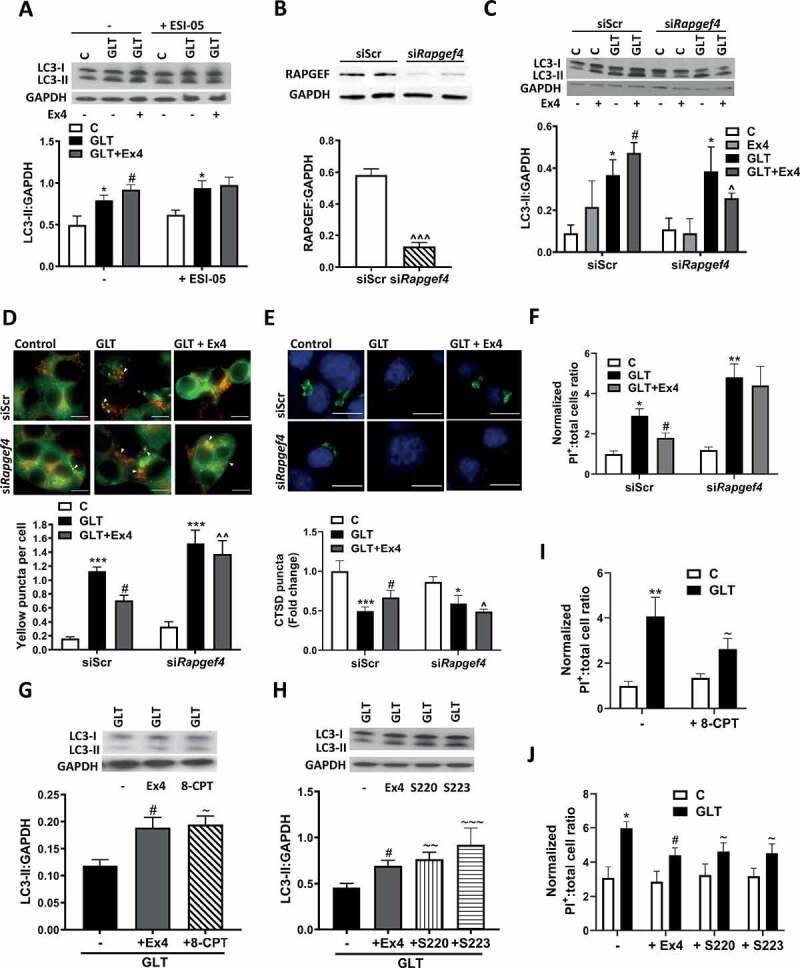
Exendin-4 stimulates autophagic flux via RAPGEF4 signaling. (A) INS-1E were pre-incubated with 10 μM ESI-05 for 1 h prior to the addition of GLT -/+ Ex4 for 6 h. LC3 was analyzed by western blotting and quantified as LC3-II:GAPDH ratio. n = 6. (B-F) INS-1E (**B, C, E and F**) or INS-1E stably expressing mCherry-GFP-LC3 (D) were treated with scrambled siRNA (siScr) or siRNA against *Rapgef4* isoform A (si*Rapgef4*) for 48 h prior to treatment with GLT -/+ Ex4 for 4 (**D**), 6 (B and C) or 18 h (E and F). (B) RAPGEF protein was analyzed by western blotting and quantified as RAPGEF:GAPDH ratio. n = 6. (C) LC3 was analyzed by western blotting and quantified as LC3-II:GAPDH ratio. n = 4. (**D**) Autophagic flux was assessed by live cell imaging of mCherry-GFP-LC3 and quantified by measurement of co-localizing red and green (yellow) puncta using Volocity software. n = 5. (E) Lysosomal function was assessed by immunostaining for CTSD (green) and puncta intensity quantified using Blobfinder software. n = 5. (F) Cell death was assessed by PI-Hoechst staining and quantified as PI-positive cells relative to total cell number. n = 3. (G-J) INS-1E were treated with GLT -/+ 100 nM Ex4, 10 μM 8-CPT-2Me-cAMP (8-CPT), 10 µM Sp-8-BnT-cAMPS (S220) or 10 µM Sp-8-BnT-2ʹ-O-Me-cAMPS (S223) for 6 h. (G and H) LC3 was analyzed by western blotting and quantified as LC3-II:GAPDH ratio. n = 5. (I and J) Cell death was assessed by PI-Hoechst staining and quantified as positive cells relative to total cell number. n = 5. Results are expressed as raw densitometry units corrected for the loading control as stated or normalized to control and expressed as fold change. All data are mean ± SEM of three to six individual experiments. Statistical analysis was performed using an unpaired t-test (**B**) or a one-way (**G and H**) or two-way ANOVA (**A, C, D, E, F, I, J**) followed by Bonferroni’s post-hoc test. *P < 0.05, **P < 0.01, ***P < 0.005 effect of GLT; #P < 0.05 effect of Ex4; ^P < 0.05, ^^P < 0.01, ^^^P < 0.005 effect of knockdown; ~P < 0.05, ~~P < 0.01, ~~~P < 0.005 effect of agonist. Scale bars: 10 µm.

Previous studies have shown that *RAPGEF4* is the predominant isoform expressed in human pancreatic β-cells with minimal expression of *RAPGEF3* [31]. We confirmed predominant expression of *RAPGEF4* over *RAPGEF3* in mouse and human β-cells using published RNA datasets (Fig. S2) [32,33]. Application of a specific RAPGEF4 inhibitor, ESI-05 [34], showed a similar effect to ESI-09 and prevented the Ex4-induced increase in LC3-II (Figure 2A). We further explored a role for RAPGEF4 by siRNA silencing. Previous knockout mouse studies have shown the RAPGEF4 isoform A to be critical for the Ex4 induced stimulation of INS secretion [35]. We therefore downregulated RAPGEF4 protein in INS-1E cells using siRNA against *Rapgef4* isoform-A (Figure 2B). There was no impact of RAPGEF4 downregulation on cAMP levels in response to Ex4 (Fig. S1B) suggesting that any impact is RAPGEF4 specific. Consistent with the RAPGEF4 inhibitor, siRNA downregulation prevented the Ex4-mediated increase in LC3-II (Figure 2C). To assess the impact on autophagic flux, we used INS-1E expressing mCherry-GFP-LC3 [18,27]. As previously described [18], treatment of INS-1E with GLT increased yellow puncta indicating an impairment in flux. Co-treatment with Ex4 partially prevented this accumulation but this effect was reversed by RAPGEF4 downregulation (Figure 2D). Consistent with these findings, while treatment with Ex4 partially prevented the GLT-induced decrease in lysosomal CTSD (cathepsin D) staining, indicative of improved lysosomal function [18], this effect was prevented by downregulation of RAPGEF4 (Figure 2E). These effects appear to be critical for the cytoprotective effects of Ex4 since RAPGEF4 downregulation prevented the Ex4-mediated decrease in GLT-induced cell death (Figure 2F). Importantly, the effect of Ex4 on both LC3-II (Figure 2G, H) and cell survival (Figure 2I, J) was mimicked by a non-isoform selective RAPGEF agonist (8-CPT-2Me-cAMP), and two RAPGEF4-specific agonists (Sp-8-BnT-cAMPS [S220] and Sp-8-BnT-2ʹ-O-Me-cAMPS [S223]) [36], further supporting a role for RAPGEF4 in this pathway.

### Exendin-4 mediated autophagy is Ca^2+^-dependent

**Figure 3.**
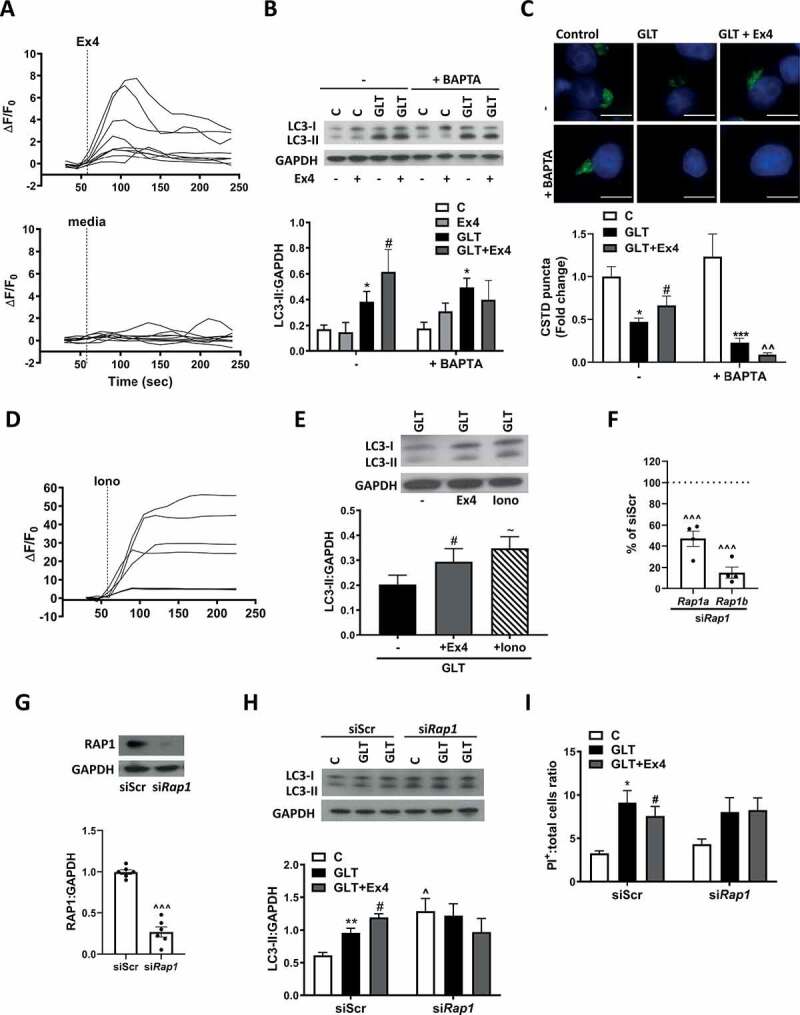
Exendin-4 stimulates autophagy via calcium signaling. (A) INS-1E were pre-incubated with 2 μM Fluo-8 AM for 35 min followed by 20 min washout with Krebs-HEPES. Cells were stimulated with either 15 mM glucose (media) or 15 mM glucose + 100 nM Ex4. Changes in fluorescence were detected by fluorescence microscopy and expressed as ΔF/F_0_. Graphs are representative of four individual experiments. (B and C) INS-1E were pre-incubated with 10 μM BAPTA-AM for 1 h prior to the addition of GLT -/+ Ex4 for either 6 (B) or 18 h (C). (**B**) LC3 was analyzed by western blotting and quantified as LC3-II:GAPDH ratio. (**C**) Lysosomal function was assessed by immunostaining for CTSD and puncta intensity quantified using Blobfinder software. Results are normalized to control and expressed as fold change. n = 6 (**B**) or 5 (**C**). (D) INS-1E were pre-incubated with 2 μM Fluo-8 AM for 35 min followed by 20 min washout with Krebs-HEPES. Cells were stimulated with 1 μM Ionomycin (Iono) and analyzed as described in (**A**). Graph is representative of three experiments. (E) INS-1E were treated with GLT -/+ Ex4 or 1 μM Ionomycin for 6 h. LC3 was analyzed by western blotting, quantified as LC3-II:GAPDH ratio and expressed relative to control. n = 6. (F-I) INS-1E were treated with scrambled siRNA (siScr) or siRNA against *Rap1a* and *Rap1b* (si*Rap1ab*) for 48 h prior to treatment with GLT -/+ Ex4 for 6 (H) or 18 h (I). (F) *Rap1* mRNA expression was analyzed by RT-PCR and quantified as either *Rap1a* or *Rap1b* corrected for *Ppia/cyclophilin a* and expressed as % of siScr control. n = 4. (G) RAP1 protein expression was analyzed by western blotting using an antibody specific for both RAP1A and RAP1B isoforms. RAP1 expression is quantified as RAP1:GAPDH ratio. n = 6. (**H**) LC3 was analyzed by western blotting and quantified as LC3-II:GAPDH ratio. n = 5. (**I**) Cell death was assessed by PI-Hoechst staining and quantified as PI positive cells relative to total cell number. n = 6. Results are expressed as raw densitometry units corrected for the loading control as stated or normalized to control and expressed as fold change. All data are mean ± SEM of three to six individual experiments. Statistical analysis was performed using an unpaired t-test (F and G), or a one-way (**E**) or two-way ANOVA (**B, C, H, I**) followed by Bonferroni’s post-hoc test. *P < 0.05, ***P < 0.005 effect of GLT; #P < 0.05 effect of Ex4; ~P < 0.05 effect of Iono, ^^^P < 0.05 effect of knockdown. Scale bars: 10 µm.

We next explored the downstream signaling pathways of RAPGEF4. Past research has shown the release of Ca^2+^ from intracellular stores to be a critical downstream component for RAPGEF4 signaling, particularly for the regulation of INS secretion by GLP1R agonists [30]. Treatment of INS-1E with Ex4 increased intracellular Ca^2+^ levels (Figure 3A, S3A, and S3B). Chelation of Ca^2+^ using BAPTA-AM prevented the Ex4-mediated increase in LC3-II (Figure 3B) and also the Ex4-mediated protection of lysosomal CTSD staining (Figure 3C). To confirm a role for Ca^2+^, we incubated INS-1E with ionomycin, which invokes Ca^2+^ release from intracellular stores [37]. As expected, ionomycin caused a dramatic increase in intracellular Ca^2+^ (Figure 3D and S3C) and also increased LC3-II compared to GLT alone (Figure 3E). These results support a role for Ca^2+^ downstream of RAPGEF4 in the Ex4-mediated stimulation of autophagy.

Existing research highlights a vital role for RAP1, a Ras-related GTPase, in intersecting RAPGEF4 with its downstream mediators including Ca^2+^signaling pathways, with RAP1 being essential for RAPGEF4-mediated INS secretion by pancreatic β-cells [38]. We silenced *Rap1* in INS-1E using siRNA against both *Rap1a* and *Rap1b* isoforms (Figure 3F, G). Under RAP1 protein downregulation, Ex4 failed to increase LC3-II levels in the presence of GLT (Figure 3H) and did not protect the cells from GLT-induced cell death (Figure 3I). However, because downregulation of RAP1 significantly increased LC3-II levels in control conditions (Figure 3H), we cannot conclusively confirm a role for RAP1 in mediating the Ca^2+^-dependent effects of Ex4 on autophagy.

### Exendin-4 mediated autophagy is independent of the AMPK and MTOR pathways

**Figure 4.**
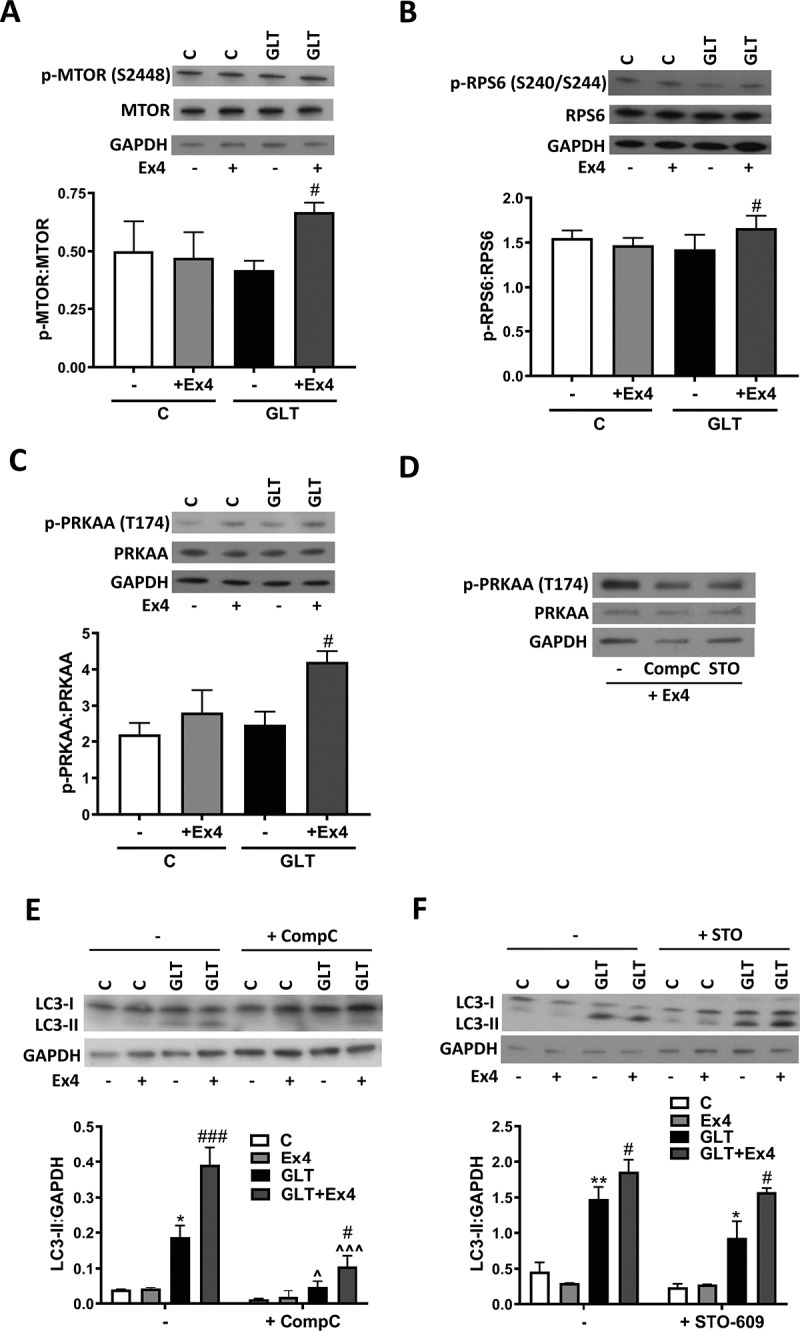
Exendin-4 stimulates autophagy independently of MTOR and AMPK signaling. (A-C) INS-1E were treated with GLT -/+ Ex4 for 2 h. Western blotting was used to quantify p-MTOR (S2448) (A), p-RPS6 (S240/244) (B) and p-PRKAA/AMPKα (T174) (C) and quantified relative to total MTOR, RPS6 or PRKAA. n = 5 (A and C) or 6 (**B**). (D) INS-1E were pre-incubated with 10 μM STO-609 or 10 μM Compound C for 1 h prior to the addition of 100 nM Ex4 for 1 h. p-PRKAA (T174) was analyzed by western blotting. Images are representative of three individual experiments. (E and F) INS-1E were pre-incubated with 10 μM STO-609 (E) or 10 μM Compound C (F) for 1 h prior to the addition of GLT -/+ Ex4 for 6 h. LC3 was analyzed by western blotting and quantified as LC3-II:GAPDH ratio. n = 3 (**E**) or 4 (**F**). Results are expressed as raw densitometry units corrected for the loading control as stated. All data are mean ± SEM of three to six individual experiments. Statistical analysis was performed using either a one-way (**A, B, C**) or two-way ANOVA (**E and F**) followed by Bonferroni’s post-hoc test. *P < 0.05, **P < 0.01 effect of GLT; #P < 0.05, ###P < 0.005 effect of Ex4; ^P < 0.05, ^^^P < 0.005 effect of inhibitor.

We next explored the potential downstream mediators of Ex4-regulated autophagy. Stimulation of INS-1E with Ex4 in the presence of GLT increased phosphorylation of the master autophagy regulator MTOR (mechanistic target of rapamycin kinase) (Figure 4A) and its downstream target RPS6 (ribosomal protein S6) (Figure 4B), consistent with activation of this pathway [2]. This is also consistent with the increase in p-AKT by Ex4 (Figure 1B), an upstream activator of MTOR. Since the active form of MTOR inhibits autophagy [2], these findings suggest that Ex4 regulates autophagy via an MTOR-independent pathway.

5ʹ AMP-activated protein kinase (AMPK) can stimulate autophagy independently of MTOR [2] and activation of the CAMKK-AMPK pathway has been shown to be critical for the activation of autophagy in cardiomyocytes by an RAPGEF activator [39]. In our model, Ex4 increased phosphorylation of AMPK at Thr174 (Figure 4C). Chemical inhibition of AMPK using compound C (Figure 4D) markedly decreased the GLT-induced increase in LC3-I to LC3-II conversion (Figure 4E). However, Ex4 continued to increase LC3-II levels in the presence of AMPK inhibition (Figure 4E). To further exclude this pathway, the upstream regulator CAMKK was inhibited using STO-609 (Figure 4D). In the presence of this inhibitor, Ex4 continued to increase LC3-II (Figure 4F). These data suggest that Ex4 mediates autophagy via a mechanism that is independent of both AMPK and MTOR.

### Signaling via PPP3/calcineurin is required for Exendin-4 mediated autophagy and *β*-cell survival

**Figure 5.**
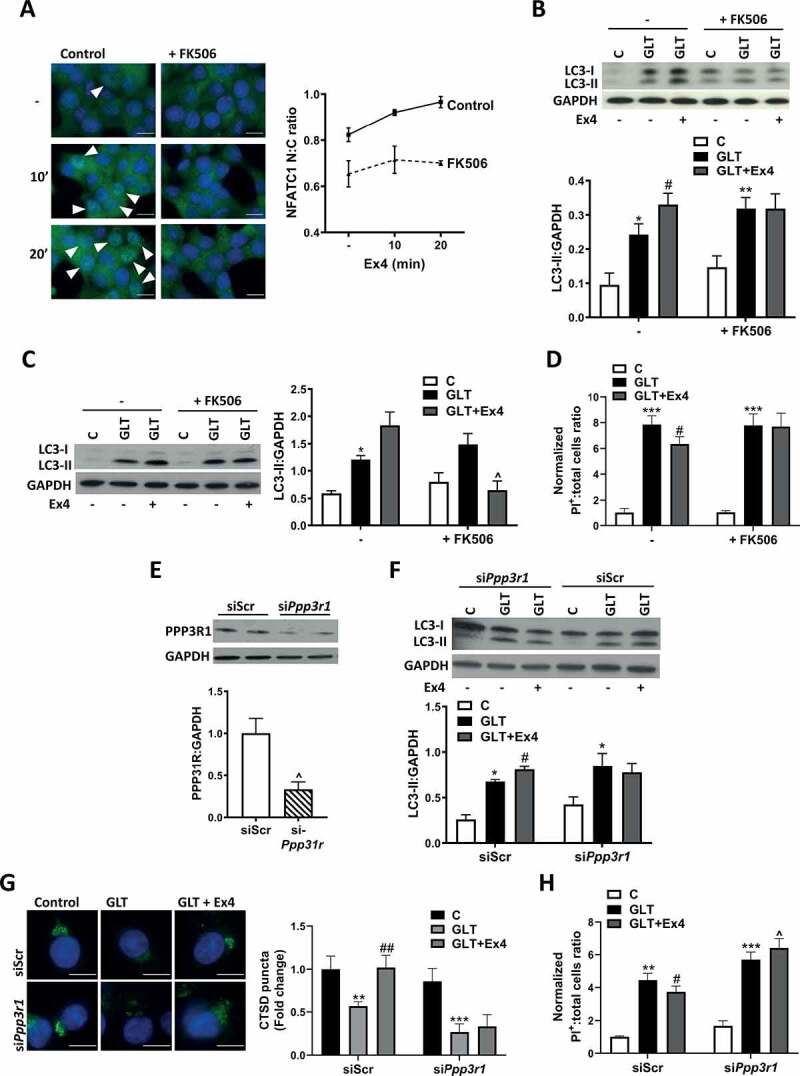
PPP3/calcineurin is essential for the cell protective effects of exendin-4. (A) INS-1E were incubated with 15 mM glucose and 100 nM Ex4, without or with 100 nM FK506 for 0, 10 or 20 min. NFATC1 translocation was visualized using immunostaining. Nuclear to cytoplasmic (N:C) ratio was quantified using ImageJ. n = 3. Images are representative of three individual experiments. (B-D) INS-1E (B and D) or human islets (C) were pre-incubated with 100 nM FK506 for 1 h prior to the addition of GLT -/+ Ex4 for 6 (B), 16 (D) or 48 h (**C**). (B and C). LC3 was analyzed by western blotting and quantified as LC3-II:GAPDH ratio. (**D**) Cell death was assessed by PI-Hoechst staining and quantified as positive cells relative to total cell number. n = 3 (**C**), 5 (**B**), 6 (**D**). (E-H) INS-1E were treated with scrambled siRNA (siScr) or siRNA against *Ppp3r1/calcineurin B1* (si*Ppp3r1*) for 48 h prior to treatment with GLT -/+ Ex4 for either 6 h (E and F) or 18 h (G and H). (E) PPP3R1 protein was analyzed by western blotting and quantified as PPP3R1:GAPDH ratio. (F) LC3 was analyzed by western blotting and quantified as LC3-II:GAPDH ratio. (G) Lysosomal function was assessed by immunostaining for CTSD and puncta intensity quantified using Blobfinder software. (H) Cell death was assessed by PI-Hoechst staining and quantified as positive cells relative to total cell number. n = 4 (**E, G, H**) or 5 (**F**). Results are expressed as raw densitometry units corrected for the loading control as stated or normalized to control and expressed as fold change. All data are mean ± SEM of three to six individual experiments. Statistical analysis was performed using an unpaired t-test (**E**), or a two-way ANOVA (**B-D, F-H**) followed by Bonferroni’s post-hoc test. *P < 0.05, **P < 0.01, ***P < 0.005 effect of GLT; #P < 0.05, ##P < 0.01 effect of Ex4; ^ P < 0.05 effect of inhibitor or knockdown. Scale bars: 10 µm.

Recent studies have uncovered a novel pathway of autophagic regulation, which involves activation of PPP3/calcineurin through the release of lysosomal Ca^2+^ stores [40]. This activation of PPP3 causes dephosphorylation of TFEB (transcription factor EB) causing its dissociation from a lysosomal complex and translocation to the nucleus where it induces transcription of genes involved in autophagy and lysosomal biogenesis [40,41]. Since we have previously shown that the effect of Ex4 on autophagic flux is mediated by an improvement in lysosomal function [18], this pathway was explored. We first determined whether Ex4 activated PPP3. Treatment of INS-1E cells caused translocation of the PPP3 downstream target, NFATC1 (nuclear factor of activated T cells 1), to the nucleus, which was prevented by a specific PPP3 inhibitor FK506 (Figure 5A). Inhibition of PPP3 using FK506 prevented the Ex4-induced increase in LC3-II in both INS-1E (Figure 5B) and in isolated human islets (Figure 5C). This was accompanied by loss of the protective effect of Ex4 over GLT-induced cell death (Figure 5D). We further explored a role for PPP3 using siRNA knockdown of *Ppp3r1/calcineurin B1* (Figure 5E), which has previously been shown to be the predominant isoform in pancreatic β-cells [42]. PPP3R1 downregulation prevented the Ex4-mediated increase in LC3-II (Figure 5F), the Ex4-mediated increase in lysosomal CTSD staining (Figure 5G) and also the protective effects of Ex4 on cell survival (Figure 5H). These data support a role for PPP3R1 in regulating Ex4-mediated autophagy.

### Exendin-4 stimulates autophagy and confers survival via TFEB

**Figure 6.**
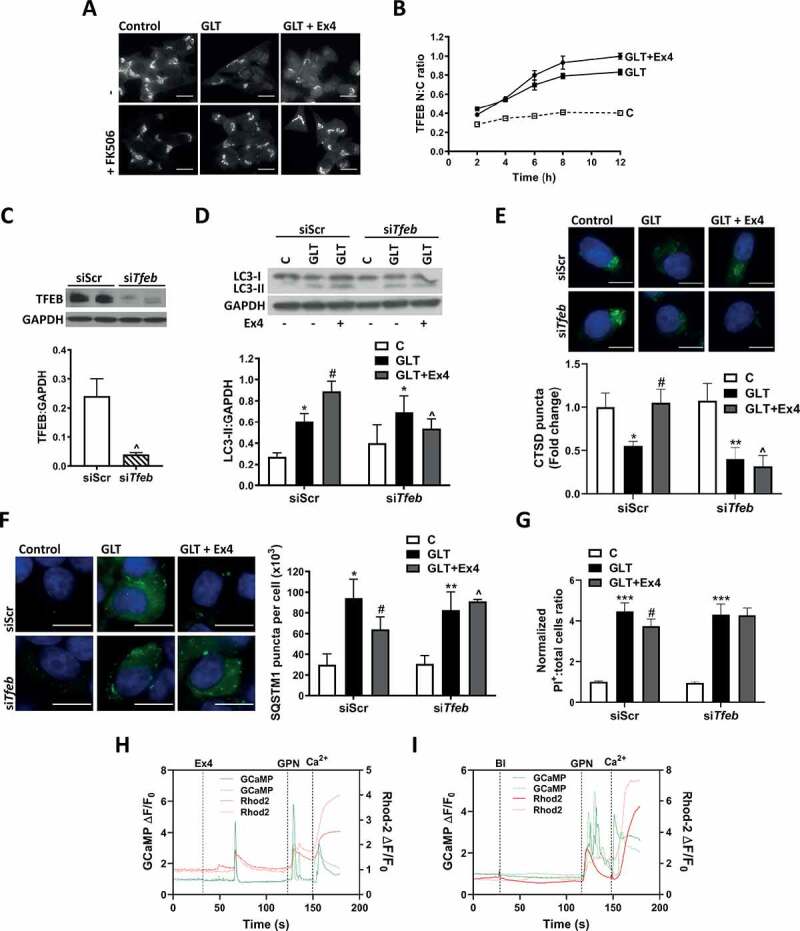
TFEB mediates the cell protective effects of exendin-4. (A and B) INS-1E were pre-incubated with 100 nM FK506 for 1 h prior to the addition of GLT -/+ Ex4 for 6 (A) or 2–12 h (B). TFEB translocation was visualized using immunostaining and quantified as nuclear to cytoplasmic (N:C) ratio using ImageJ. (**A**) Results are representative of three individual experiments. (**B**) Mean ± SEM of two to four individual experiments. (C-G) INS-1E were treated with scrambled siRNA (siScr) or siRNA against *Tfeb* (si*Tfeb*) for 48 h prior to treatment with GLT -/+ Ex4 for either 6 (C and D) or 18 h (E-G). (C) TFEB protein was analyzed by western blotting and quantified as TFEB:GAPDH ratio. (D) LC3 was analyzed by western blotting and quantified as LC3-II:GAPDH ratio. (E) Lysosomal function was assessed by immunostaining for CTSD and puncta intensity quantified using Blobfinder software. (F) Autophagic flux was assessed by immunostaining for SQSTM1 and puncta intensity quantified using Blobfinder software. (G) Cell death was assessed by PI-Hoechst staining and quantified as positive cells relative to total cell number. n = 4 (**C, D, F, G**) or 5 (**E**). (H and I) INS-1E were transfected with GCaMP3-ML1 (GCaMP) for 24 h prior to addition of Rhod-2-AM (Rhod2) for 30 min followed by 30 min washout in Tyrode’s solution at 11 mM glucose. Cells were stimulated with 100 nM Ex4 (H) or media only (Bl) (I) for 90 s. Specificity of the construct was confirmed by addition of 400 μM glycyl-L-phenylalanine-beta-naphthylamide (GPN) followed by 66 mM CaCl_2_ (Ca^2+^). Changes in fluorescence at Ex:490, Em:525 (for GCaMP) and Ex:550, Em:580 (for Rhod-2-AM) were detected by confocal microscopy (Nikon A1R) and expressed as ΔF/F_0_. Data is representative of five individual experiments. Results are expressed as raw densitometry units corrected for the loading control as stated or normalized to control and expressed as fold change. All data are mean ± SEM of three to five individual experiments. Statistical analysis was performed using an unpaired t-test (**C**), or a two-way ANOVA (D-G) followed by Bonferroni’s post-hoc test. * P < 0.05, **P < 0.01, ***P < 0.005 effect of GLT; #P < 0.05 effect of Ex4; ^P < 0.05 effect of knockdown. Scale bars: 10 µm.

In our previous study [18], we showed that treatment of β-cells with Ex4 increased TFEB translocation to the nucleus, which was accompanied by increased transcription of TFEB target genes. We confirmed in this study that the Ex4-mediated increase in TFEB translocation was inhibited by the PPP3 inhibitor FK506 (Figure 6A). Time course studies showed that co-treatment with Ex4 caused a time-dependent increase in TFEB translocation in comparison to GLT alone, with maximal effect at >8 h treatment (Figure 6B). To test whether TFEB is essential for Ex4-mediated autophagy, we downregulated TFEB protein expression using siRNA (Figure 6C). TFEB downregulation prevented the Ex4-mediated increase in LC3-II (Figure 6D) and the increase in lysosomal CTSD staining (Figure 6E). TFEB downregulation also prevented the Ex4-mediated decrease in SQSTM1 accumulation, a marker of deregulated autophagic flux (Figure 6F) and prevented the protective effects of Ex4 on cell survival (Figure 6G). This data supports a role for TFEB in regulating autophagic flux and mediating the cell-protective effects of Ex4.

We next tested whether Ex4 caused release of lysosomal Ca^2+^ since this intracellular source has been shown to be critical for the activation of TFEB [40]. Using a Ca^2+^ sensor that localizes to the lysosomal membrane [43], we revealed that treatment with Ex4 increased Ca^2+^ around the lysosomal membrane evidenced by a spike in GCaMP3-ML1 signal, consistent with release of Ca^2+^ from this compartment (Figure 6H, I, and S4). Ex4 also caused release of Ca^2+^ from the endoplasmic reticulum evidenced by a decrease in ER-GCaMP6-150 [44] signal (Fig. S5) as well increased Ca^2+^ intake across the plasma membrane evidenced by a spike in pGP-CMV-GCaMP6s [45] signal (Fig. S6). The effect of Ex4 on the movement of Ca^2+^ occurred 25–35 s following the addition of Ex4 and was simultaneous with an increase in total intracellular Ca^2+^ as assessed using Rhod-2 [46].

### TFEB overexpression protects *β*-cells from glucolipotoxicity-induced cell death

**Figure 7.**
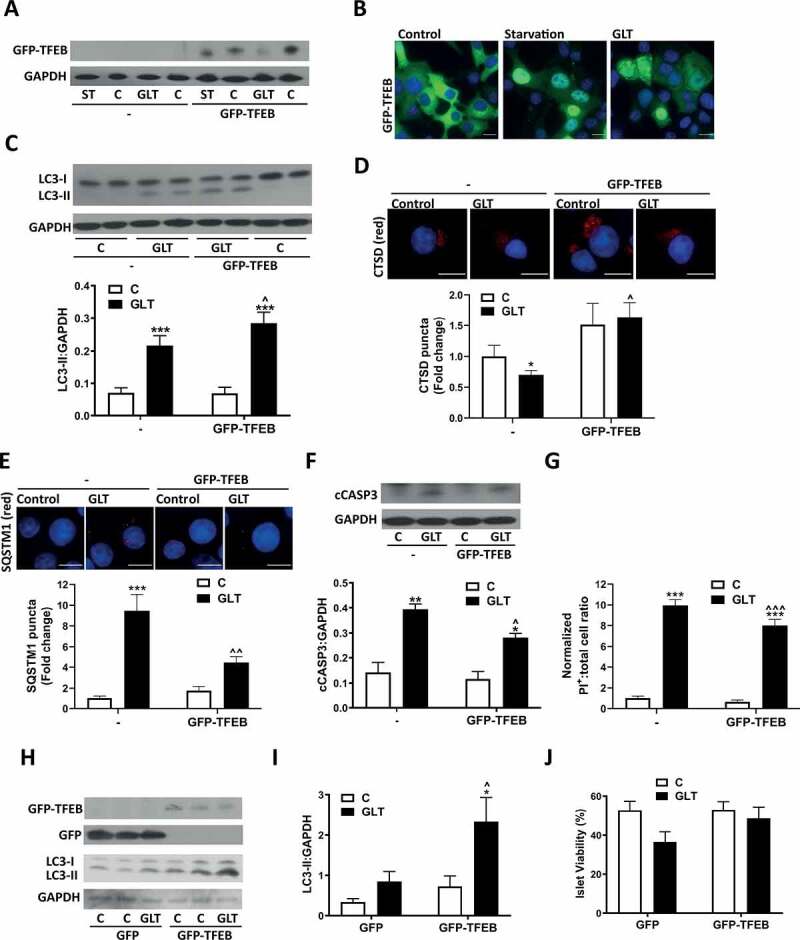
TFEB overexpression protects β-cells from GLT-mediated cell death. (A-G) Stable INS-1E clones that were non-expressing (-) or GFP-TFEB-expressing (GFP-TFEB) cells were starved (ST) for 2 h in HBSS (A and B) or incubated with GLT for 6 (**A-C, F**) or 18 h (**D, E and G**). (A) Expression of GFP-TFEB was confirmed by western blotting for GFP. The differences in migration pattern upon starvation or addition of GLT represent dephosphorylation of TFEB, which alters the migration rate of the protein [40]. (B) GFP-TFEB translocation was confirmed in response to both starvation and GLT exposure. Images are representative of three individual experiments. (C) LC3 was analyzed by western blotting and quantified as LC3-II:GAPDH ratio. (D) Lysosomal function was assessed by immunostaining for CTSD and puncta intensity quantified using Blobfinder software. (E) Autophagic flux was assessed by immunostaining for SQSTM1 and puncta intensity quantified using Blobfinder software. (F) Cell death was assessed by western blotting for cCASP3. (G) Cell death was assessed by PI-Hoechst staining and quantified as positive cells relative to total cell number. n = 4 (D and E), 5 (**F**) or 8 (C and G). (H-J) Human islets were transfected with GFP-only (GFP) or pEGFP-N1-TFEB (GFP-TFEB) and islets cultured for 48 h before exposure to GLT for a further 48 h. (H) GFP expression and LC3 were analyzed by western blotting. (I) LC3 was quantified as LC3-II:GAPDH ratio. (J) Cell death was assessed by PI-Hoechst staining and quantified as % viability. n = 4 (**J**) or 5 (H and I). Results are expressed as raw densitometry units corrected for the loading control as stated or normalized to control and expressed as fold change. All data are mean ± SEM of four to eight individual experiments. Statistical analysis was performed using a two-way ANOVA followed by Bonferroni’s post-hoc test *P < 0.05, **P < 0.01, ***P < 0.005 effect of GLT; ^P < 0.05, ^^P < 0.01, ^^^P < 0.005 effect of overexpression. Scale bars: 10 µm.

Independent studies have shown that activation of TFEB can provide protection from the effects of the metabolic syndrome by improving the capacity of the cell to upregulate autophagy [47,48]. We therefore determined whether overexpression of TFEB was able to protect β-cells from the cytotoxic effects of GLT treatment. We generated a stable INS-1E cell line expressing GFP-TFEB (Figure 7A). GFP-TFEB translocated to the nucleus upon acute nutrient starvation and in response to GLT (Figure 7B), similar to that of endogenous protein [18]. GFP-TFEB expressing cells showed a greater increase in LC3-II in response to GLT compared to control non-GFP-TFEB expressing cells (Figure 7C). Similarly, expression of GFP-TFEB increased lysosomal CTSD staining and also prevented the GLT-induced decrease in CTSD (Figure 7D). Assessment of autophagic flux using a measure of SQSTM1 accumulation showed that expression of GFP-TFEB was able to partially prevent the impairment in autophagic flux induced by GLT, as demonstrated by decreased SQSTM1 accumulation (Figure 7E). Further to this, GFP-TFEB expression conferred improved cell survival when cells were challenged with GLT as evidenced by a decrease in the apoptotic marker cleaved CASP3 (Figure 7F) and also decreased total cell death measured via PI staining (Figure 7G). Expression of GFP-TFEB in human islets (Figure 7H) also improved the autophagic capacity of the islet as determined by increased LC3-I to LC3-II conversion in response to GLT (Figure 7I) and also decreased GLT-induced cell death (Figure 7J).

### Exendin-4 increases lysosomal marker expression in a mouse model of diabetes

**Figure 8.**
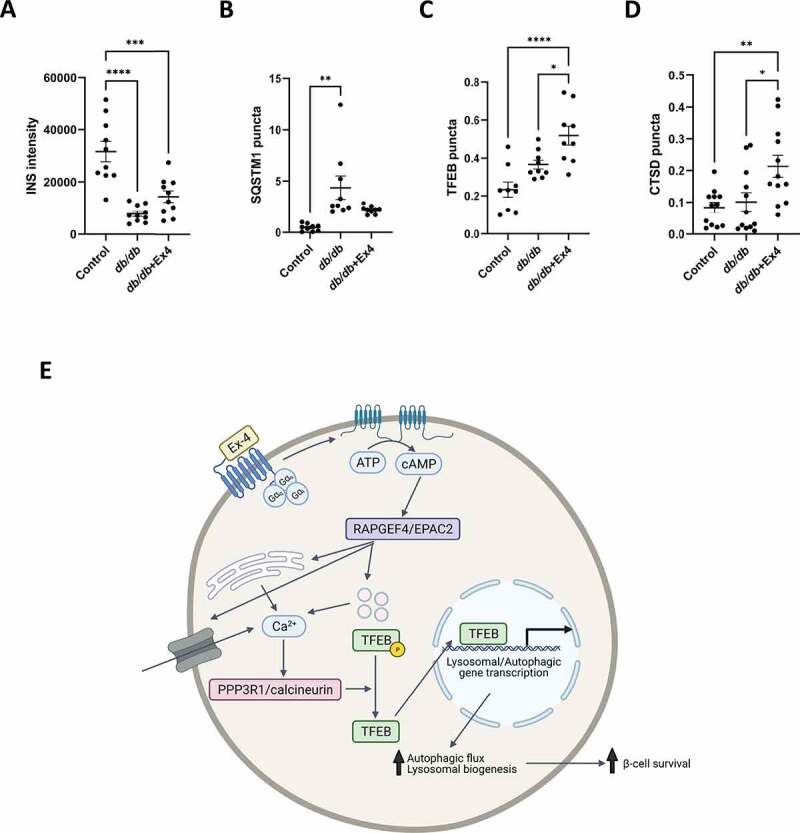
Exendin-4 increases lysosomal marker expression *in vivo*. (A-D) *db/db* mice were injected twice daily with either saline or exendin-4(1–39) at 25 nmol/kg bw for 21 d as described in [49]. Aged matched lean control C57Bl/6 J mice treated with saline were used as non-diabetic controls. Pancreatic tissue was stained for INS (A), SQSTM1 (B), TFEB (C) and CTSD (D) and imaged using confocal microscopy. Results are expressed either as raw intensity (**A**) or corrected for INS positive area (B-D). n = 5 animals, 10–15 islets imaged per condition. Statistical analysis was performed using one-way ANOVA followed by Bonferroni’s post-hoc test. * P < 0.05, ** P < 0.01, *** P < 0.005, **** P < 0.001. (E) Schematic representation of model. Ex4 activates ADCY (adenylate cyclase) through its G-protein coupled receptor which leads to the activation of cAMP. The downstream activation of RAPGEF4 then leads to an increase in intracellular Ca^2+^ concentration, which in turn stimulates PPP3R1/calcineurin activity and downstream dephosphorylation of TFEB. This causes translocation of TFEB to the nucleus and subsequent upregulated transcription of genes involved in autophagy and lysosomal biogenesis and upregulation of autophagic flux. Image created by Biorender.com.

To confirm that the mechanism uncovered in our *in vitro* study is relevant *in vivo*, we explored the impact of Ex4 treatment on autophagic and lysosomal markers in pancreatic islets from *db/db* mice, a rodent model of T2D. *db/db* mice were injected twice daily with Ex4 (25 nmol/kg body weight) for 21 d, with lean non-diabetic C57Bl/6 J mice used as non-diabetic controls. Glucose and INS tolerance data from this study has previously been published and shows that Ex4 treatment improved glucose tolerance of the *db/db* mice (Figures 3 and Figure 4 in [49]). Immunofluorescence staining of pancreatic tissue from these mice showed that the *db/db* model had lower INS protein expression compared to the lean controls (Figure 8A, and S7), consistent with β-cell failure characteristic of this model. Islets from *db/db* mice also showed increased SQSTM1 accumulation within pancreatic islets suggestive of an impairment in autophagic flux (Figure 8B, and S7A). There was no significant change in TFEB or CTSD protein expression (Figure 8C, D, S7B, and S7C). Treatment of *db/db* mice with Ex4 showed a trend toward increased INS protein expression (Figure 8A), consistent with the improved glucose tolerance. Ex4 treatment increased both TFEB and CTSD protein expression (Figure 8C, D), suggestive of improved lysosomal function. There was a trend toward decreased SQSTM1 puncta following treatment with Ex4 although this effect was not significant (Figure 8B).

### Discussion

The maintenance of a functional pancreatic β-cell mass is essential to prevent the inexorable progression of T2D toward the need for injected INS replacement and increased risk of the complications of chronic high glucose levels. Understanding how β-cell mass can be preserved is essential to enable identification of novel target pathways for the treatment of T2D. Autophagy has been recognized as a potential target pathway since studies have shown that improving autophagic capacity can improve both β-cell function and mass in models of T2D [21,22]. In the current study, we identify the RAPGEF4-Ca^2+^-PPP3/calcineurin-TFEB axis as a potential target to improve autophagic flux and β-cell survival (Figure 8E).

There are several independent reports of GLP1R agonists stimulating autophagy, both in pancreatic β-cells and other tissue types [18,24–26] but little detail has been provided on the underlying mechanisms. A recent study reported that treatment of mice with a DPP-4 inhibitor, which exerts its anti-diabetic actions via inhibition of the rapid degradation of endogenous GLP-1 (glucagon like peptide 1), decreased phosphorylation of MTOR suggesting inhibition of MTOR activity and subsequent stimulation of autophagy [26]. In our model system, treatment with Ex4 increased MTOR signaling, consistent with the current dogma that GLP1R agonists stimulate the MTOR pathway to promote proliferation [50,51] but does not support a role for MTOR in mediating GLP1R agonist-stimulated autophagy. Another study reported a potential role for AMPK in the regulation of autophagy by the GLP1R agonist liraglutide [52] whereby it was proposed that liraglutide stimulated autophagy via the inhibition of AMPK. This is surprising because activation of AMPK usually stimulates autophagy [2]. In our study, we show that treatment of INS-1E cells with Ex4 did not inhibit AMPK but rather caused a small stimulation in phosphorylated AMPK. Furthermore, inhibition of AMPK using a chemical inhibitor did not prevent the Ex4-mediated increase in LC3-II. However, it should be noted that this inhibitor caused a marked decrease in the GLT-induced increase in LC3-II, which made this analysis difficult and prevents complete exclusion of this pathway.

The pro-survival effects of GLP1R agonists in β-cells have largely been attributed to signaling via the AKT-MTOR axis through inhibition of apoptosis although a role for cAMP-dependent mechanisms have also been reported [28,29]. Our data excludes a role for AKT, MTOR and PRKA in mediating Ex4-induced autophagy but identifies a novel role for RAPGEF4 in mediating Ex4-induced autophagy and its pro-survival effects. While RAPGEF4 is known to be critical for the potentiation of glucose-induced INS secretion by GLP-1 in β-cells [28,30], its cyto-protective role has largely been unexplored, unlike in the heart where the role for RAPGEF3 is well described [53]. Here, we provide strong evidence for RAPGEF4-mediated regulation of autophagy and cell survival as indicated by: i) prevention of Ex4-mediated autophagy in INS-1E by downregulation of RAPGEF4; ii) inhibition of Ex4-mediated autophagy by RAPGEF inhibitors in INS1E and in mouse and human islets; iii) stimulation of autophagy and increased cell survival in INS-1E by RAPGEF4 specific agonists. These findings are consistent with the induction of autophagy by an RAPGEF agonist in cardiomyocytes [39]. However, RAPGEF-regulated autophagy appears complex since signaling through this pathway has been shown to inhibit autophagy in response to alpha-hemolysin via activation of CAPN (calpain) [54] as well as during chronic stimulation of β-cells with oleate where RAPGEF4 inhibition was necessary for the oleate-mediated increase in autophagy [55]. These studies parallel the complex regulation of autophagy by its downstream mediator Ca^2+^ [56] and demonstrates that the regulation of autophagy by this pathway appears to be both cell-type and stimulus specific, and thus warrants careful examination. It should be noted that while our data supports a role for the RAPGEF4 isoform in the Ca^2+^-PPP3/calcineurin-TFEB axis, the use of ESI-09 does not allow us to identify the RAPGEF isoform in the human islets studies since this inhibitor acts on both RAPGEF3 and RAPGEF4. However, using existing RNA datasets of mouse and human origin [32,33], we replicate previous studies in human islets [31] suggesting that *RAPGEF4* is the predominant isoform in pancreatic β-cells, with little expression of *RAPGEF3*. This is consistent with our data showing that the Ex4-dependent effects on autophagy are prevented by the RAPGEF4 selective inhibitor ESI-05 and can be mimicked by RAPGEF4 selective agonists. Combined, these findings support a role for RAPGEF4 rather than RAPGEF3 in this mechanism.

After excluding the canonical MTOR and AMPK signaling pathways, we uncovered a novel role for the PPP3/calcineurin-TFEB axis in the Ex4-mediated stimulation of autophagy. Previous studies have reported activation of this pathway by starvation and exercise [40,41] via a mechanism that involves the release of lysosomal Ca^2+^, which activates PPP3 and subsequently TFEB localized to this compartment [40]. Some studies have reported upregulation of the PPP3-TFEB pathway in the absence of changes to lysosomal Ca^2+^suggesting that an increase in total intracellular Ca^2+^ may be sufficient [47]. Our data showed that treatment of INS-1E cells with Ex4 increased intracellular Ca^2+^, which was associated with release of endoplasmic reticulum Ca^2+^ stores, increased uptake of Ca^2+^ across the plasma membrane and also increased movement of Ca^2+^ across the lysosomal membrane. This may represent release of Ca^2+^ from the lysosomal compartment but could also represent refilling of this compartment [43]. The finding that Ex4 increases the localization of Ca^2+^ around the lysosomes is consistent with activation of TFEB via the previously described mechanism [40]. Studies in β-cells have reported activation of PPP3 and its downstream target NFAT by GLP-1 [57]. However, a recent study in human islets isolated from either juvenile or adult donors showed that while Ex4 stimulated proliferation via a PPP3-mediated pathway in juvenile islets, this effect was absent in adult islets [58]. In the current study, we confirmed that PPP3 was essential for the Ex4-mediated increase in autophagy in human islets isolated from adult donors using a specific PPP3 inhibitor. It may be that while the downstream pathway of NFAT activation, which serves to stimulate cellular proliferation is lost in the adult islets, the upstream activation of PPP3 may be retained, consistent with the preservation of the GLP-1 signaling pathways influencing INS secretion in the adult model [58]. It should be noted that in a model of tacrolimus (also designated FK506) -induced diabetes, Ex4 continued to promote cell survival despite continued PPP3 inhibition [59]. In this *in vivo* model, Ex4 increased β-cell survival through improvements in lysosomal function, which promoted autophagosomal-lysosomal fusion. Interestingly, in this model Ex4 failed to stimulate autophagy, which is consistent with the inhibition of Ex4-mediated autophagic stimulation by PPP3 inhibition in our model (Figure 5D, F) although in our hands this blockage also prevented the Ex4-mediated improvement in lysosomal staining. It is likely that the ability of Ex4 to act on the autophagy-lysosomal cascade is dependent on the external stresses deregulating the pathway, i.e. tacrolimus vs chronic GLT. It may be that Ex4 improves lysosomal function via a PPP3-independent mechanism, in addition the PPP3-TFEB axis uncovered in our model.

The lysosomal transcription factor TFEB is essential for the appropriate regulation of autophagic flux and lysosomal biogenesis [40,41]. In the current study using both siRNA downregulation of endogenous TFEB and overexpression of TFEB, we provide persuasive evidence highlighting an essential role for TFEB in Ex4-mediated autophagy and its pro-survival effects. These data are consistent with a previous study showing that overexpression of TFEB partially prevented palmitate-induced β-cell death [60] and a recent study linking GLP-1 signaling with TFEB regulation in hepatocytes [61]. Importantly, we have confirmed that expression of TFEB and its downstream target CTSD are upregulated following treatment of *db/db* mice with Ex4 *in vivo*, which correlated with a trend toward improved autophagic clearance of SQSTM1. We have also shown that TFEB overexpression exerts protective effects in human islets. These studies were complicated by the low viability of human islets in all conditions due to the complexity and duration of the study. However, consistent and reproducible data were attained over a number of independent experiments showing that TFEB overexpression in human islets stimulated autophagy and prevented GLT-induced cell death. A number of commercially available chemicals have been identified to induce TFEB activation in HeLa cells [47]. However, we were unable to detect any effect of digoxin, ikarugamycin or alexidine dihydrochloride on TFEB localization in our INS-1E model at concentrations that are not cytotoxic (results not shown). In an independent study, a novel TFEB activator improved the glucose tolerance and metabolic profile in genetically induced diabetes (*ob/ob*) and in diet-induced obesity mouse models [48]. However, the impact of this activator on β-cell function and survival remains to be fully explored. This is particularly relevant given the recent reports of decreased TFEB nuclear localization in pancreatic islets from patients with T2D [62]. The finding that improving lysosomal function using targeted nanoparticles has positive effects on β-cell function in a model of nutrient excess [63], supports the targeting of lysosomal function or biogenesis to improve lipid handling in T2D.

To conclude, we provide substantial evidence for a role of the RAPGEF4-Ca^2+^-PPP3-TFEB axis in the regulation of pancreatic β-cell autophagy and the pro-survival effects of Ex4. Importantly, we provide confirmation that these pathways are conserved in adult human islets, supporting novel therapeutic approaches targeting this pathway to preserve a functional pancreatic β-cell mass in T2D. Given the accumulating evidence supporting the application of GLP1R agonists for the treatment of other disease states, including other metabolic diseases and numerous neurological disorders [64] via mechanisms involving autophagy [61,65,66], these findings could have implications beyond the treatment of T2D.

## Materials and methods

### Cell culture

INS-1E cells were cultured in RPMI-1640 media (Sigma Aldrich, R8758) containing 50 μM β-mercaptoethanol (Sigma Aldrich, M3148), 1 mM sodium pyruvate (Sigma Aldrich, S8636), 50 U/ml penicillin, 50 μg/ml streptomycin (Sigma Aldrich, P4333) and 5% FBS (Thermo Fisher Scientific, 11550356). Palmitate:BSA was prepared as in [18]. Cells were incubated with appropriate inhibitors or agonists as in Table S1.

For siRNA silencing INS-1E cells were transfected with appropriate siRNA against *Rapgef4 isoform A* (GE Healthcare, L-101689-02-0005), *Rap1a* and *Rap1b* (Sigma Aldrich, SASI_RN02_00198438, SASI_RN02_00114089), *Ppp3r1/calcineurin B1* (Sigma Aldrich, SASI_Rn01_00105944) and *Tfeb* (Sigma Aldrich, SASI_Rn01_00063599) for 48 h using either Lipofectamine® RNAiMAX (Thermo Fisher Scientific, 13778030) or Mission siRNA transfection reagent (Sigma Aldrich, S1452) according to the manufacturer’s instructions. For assessment of autophagic flux, INS-1E cells stably expressing pBABE-puro mCherry-EGFP-LC3B (a gift from Jayanta Debnath [64] [Addgene, 22418]) were generated and imaged as previously described [18]. For assessment of TFEB overexpression, INS-1E cells were transfected with pEGFP-N1-TFEB (a gift from Shawn Ferguson [67] [Addgene, 38119]) and non-expressing (-) or expressing (+) cells selected using FACS cell sorting for GFP. INS-1E cells expressing GFP-TFEB were cultured in 400 μg/ml G418 (Alfa Aesar, J63871.AD) to maintain selection.

### Human islets

Human islets were isolated from 8 adult non-diabetic donors in either the Clinical Islet Lab, University of Alberta, Canada; the Islet Isolation Center, University of Edinburgh; The Islet Isolation Facility, University of Oxford or the Newcastle University Transplant Regenerative Medicine Laboratory, with appropriate ethical approval. All procedures were fully compliant with the declaration of Helsinki 2013: the ethical principles for medical research involving human subjects. The clinical data for associated with each islet preparation are summarized in Table S2. Islets were maintained in CMRL media (GE Healthcare, 15–110) containing 0.5% human serum albumin (Sigma Aldrich, H3667), 50 U/ml penicillin and 50 μg/ml streptomycin (Sigma Aldrich, P4333) for 24 h prior to treatment. For expression of GFP-TFEB, human islets (500–1000 per condition) were washed with PBS (Sigma Aldrich, P4417) before incubation with 0.5x trypsin-EDTA solution (Sigma Aldrich, T3924) for 3 min at 37°C. After washing and resuspension in 2.5 ml of RPMI-1640 media (Sigma Aldrich, R8758) with 1% FBS (Thermo Fisher Scientific, 11550356), 500 μl of DNA:lipofectamine complex (5 μg DNA to 15 μl Lipofectamine 2000 [Thermo Fisher Scientific, 11668027]) containing either GFP-TFEB [28] or the GFP-only vector (pEGFP-N1: Clontech, PT3027-5) was added to each well for 16 h. Media was then replaced by CMRL media for 36 h prior to 48 h treatment.

### Rodent islet isolation

Islets were isolated from 8 to 10 week old C57BL/6 mice as previously described [18]. All procedures conformed to Home Office Regulations and approved by Newcastle University Ethical Committee.

### In vivo studies

The methodology for the *in vivo* studies has previously been published [49]. Nine week old homozygous C57Bl/KsJ *db/db* mice received twice daily intraperitoneal injections of either 0.9% (w:v) sodium chloride (*db/db* saline controls and C57Bl/6 J lean controls), or Ex4(1–39) (25 nmol/kg body weight) for 21 d. Pancreatic tissues were fixed in 4% paraformaldehyde for 48 h before embedding in paraffin wax. Tissue was sectioned (4-µm thickness) prior to staining.

### Cell death measurements

For PI-Hoechst staining, cells were incubated with 10 µg/ml propidium iodide (Sigma Aldrich, P4864) and 10 µg/ml Hoechst-33342 (Sigma Aldrich, 14533) for 20 min. Images were taken with a Nikon TE2000 (×20). For INS-1E, >1000 cells were counted per condition and analysis performed using ImageJ software (NIH). Data is expressed as number of PI-positive cells per total cells (Hoechst positive). For human islets, 10 islets were imaged per condition and the percentage viability of the total islet area quantified using ImageJ software. Apoptosis was determined by western blotting for cleaved CASP3.

### Western blotting

Proteins were fractionated using 4–20% SDS-PAGE gels (Bio-Rad, 4568096, 4568093) and transferred to PVDF (Fisher Scientific, 10078559). Membranes were probed with primary antibody at 4°C overnight. The primary antibodies used in this study included anti-p-AKT (Ser473; Cell Signaling Technology, 4060), anti-AKT (Cell Signaling Technology, 4691), anti-p-PRKAA/AMPKα (Thr172; Cell Signaling Technology, 2531), anti-PRKAA/AMPKα (Cell Signaling Technology, 2532), PPP3/calcineurin (Novus Biologicals, 212306), anti-cleaved CASP3 (Cell Signaling Technology, 9661), anti-p-CREB (Ser133; Cell Signaling Technology, 9198), anti-CREB (Cell Signaling Technology, 9197), anti-RAPGEF (Cell Signaling Technology, 4156), anti-GAPDH (Hytest, 5G4), anti-GFP (Santa Cruz Biotechnology, sc-9996), anti-LC3 (Sigma Aldrich, L7543), anti-p-MTOR (Ser2448; Cell Signaling Technology, 2971), anti-MTOR (Cell Signaling Technology, 2972), anti-RAP1 (Cell Signaling Technology, 2399), anti-p-RPS6 (Ser240/244; Cell Signaling Technology, 2215), anti-RPS6 (Cell Signaling Technology, 2217) and anti-TFEB (Bethyl Laboratories, A303). After incubation with secondary antibody conjugated to horseradish peroxidase (Agilent Dako, P044801; Thermo Fisher Scientific, 61–6520), bands were detected using enhanced chemiluminescence. Immunoblots were scanned using the Bio-Rad GS-800 calibrated densitometer. Blot oversaturation was excluded by analysis with Quantity-one (Bio-Rad) software and Fiji (ImageJ) software and blots deemed to be within the linear range were used for quantification.

### Real time RT-PCR

RNA was extracted using TRIzol (Thermo Fisher Scientific, 15,596,026), and cDNA was synthesized from 1 µg RNA with Maloney murine leukemia virus (Promega Corporation, M1705). Real-time RT-PCR was performed as in [68] using custom-designed primers (Table S3). Relative mRNA levels were calculated by delta cycle threshold, corrected for *Ppia*/*cyclophilin a*, and expressed relative to siRNA scrambled control.

### Immunostaining

For staining of cells, INS-1E were plated onto coverslips were fixed with 4% paraformaldehyde and immunostaining performed as in [18]. The primary antibodies used in this study included anti-CTSD (Santa Cruz Biotechnologies, SC-6487), anti-NFATC1 (BD Pharmingen, 556602) and anti-SQSTM1 (Progen, GP62-C). Cells were imaged using a Nikon TE2000 (×100). Lysosomal puncta as determined by CTSD puncta were quantified using Blobfinder software (Uppsala University, Sweden). TFEB and NFATC1 translocation were quantified by measurement of the mean pixel intensity for the nuclear and cytoplasmic compartments using ImageJ and the nuclear:cytoplasmic ratio calculated. For these calculations, >5 images were taken per condition for each independent experiment.

For immunostaining of tissue, indirect immunofluorescence staining was carried out as in [18]. The primary antibodies used in this study included anti-CTSD (Proteintech, 21327-1-AP), anti-INS (Dako, A0564), anti-SQSTM1 and anti-TFEB (Bethyl Laboratories, A303). Tissue was imaged using a Nikon Eclipse TE2000-S and 10–15 islets imaged per condition. The intensity of INS staining and SQSTM1, TFEB and CTSD puncta within INS positive areas was quantified using FIJI software.

### cAMP determination

INS-1E cells were washed with Krebs-Ringer Buffer (135 mM NaCl, 5 mM KCl, 1 mM MgSO_4_, 0.4 mM K_2_HPO_4_, 5.5 mM glucose (VWR, 10117), 20 mM HEPES (Thermo Fisher Scientific, 15630–056), pH4.7) and pre-incubated in buffer for 30 min. Following further washing, cells were incubated at 16.7 mM glucose in the presence of 500 µM IBMX (Enzo Life Sciences, BML-PD140-0200) with or without 100 nM Ex4 for 15 min before extraction into 200 µl of 0.1 mM HCl-0.5% Triton X-100 (Sigma Aldrich, X100). cAMP was determined using the Direct cAMP ELISA kit (Enzo Life Sciences, ADI-900-066) accordingly to the manufacturer’s instructions.

### Ca^2+^ imaging

For total cell Ca^2+^ imaging, INS-1E cells were plated in black-walled, clear bottomed 96-well plates (Screenstar; Greiner-Bioone, 655866). After 24 h cells were loaded with 2 μM Fluo-8 AM (Abcam, ab142773) for 35 min, followed by 20 min washout with Krebs-HEPES-Ca^2+^ buffer (119 mM NaCl, 4.74 mM KCl, 1.19 mM MgCl_2_, 2.54 mM CaCl_2_, 1.10 mM KH_2_PO_4_, 25 mM NaHCO_3_, 10 mM HEPES, pH 7.4). Fluorescence was imaged (Ex: 490, Em:525) using a Nikon Eclipse TE2000-S every 15 s for 4 min. For each image, 10 cells were selected and Ca^2+^ transients calculated as ΔF/F_0_ using ImageJ.

For organelle specific Ca^2+^ imaging, vectors expressing GCaMP3 targeted toward lysosome (GCaMP3-ML1) [43], endoplasmic reticulum (ER-GCaMP6-150, a gift from Timothy Ryan [Addgene, 86918] [44]) and plasma membrane (pGP-CMV-GCaMP6s, a gift from Douglas Kim & GENIE Project [Addgene, 40753] [45]) were used. INS-1E were plated in cell culture slides (Cellview; Greiner-Bioone, 543079) and transfected with the specified vector using lipofectamine 2000 for 24 h. Cells were incubated with 2 μM Rhod-2-AM (Thermo Fisher Scientific, R1245MP) diluted in 10% pluronic F-127 (Sigma Aldrich, P2443) for 30 min followed by a 30 min washout in Tyrode’s solution (137 mM NaCl, 2.7 mM KCl, 1 mM MgCl_2_, 1.8 mM CaCl_2_, 0.2 mM Na_2_HPO_4_, 12 mM NaHCO_3_, 11 mM glucose, pH 7.4). INS-1E were imaged using a Nikon A1R confocal microscope for 3 min, with readings every 0.5 s at Ex:490, Em:525 (for GCaMP) and Ex:550, Em:580 (for Rhod-2-AM). 3–10 cells were selected per image and Ca^2+^ transients calculated as ΔF/F_0_ using FIJI software.

### Statistical analysis

Results are expressed as means ± SEM for the number of experimental replicates (minimum of three, maximum of eight). Statistical analysis was performed using GraphPad Prism using an unpaired Student’s two-tailed *t*-test, by one-way ANOVA using mixed effects analysis comparing all treatments or by ordinary two-way ANOVA comparing all treatments, as stated in the legend. ANOVA analysis were followed by Bonferroni’s multiple comparison test. P < 0.05 was deemed to be statistically significant.

## Supplementary Material

Supplemental Material
