# Supplementary material for: Exendin-4 stimulates autophagy in pancreatic β-cells via the RAPGEF/EPAC-Ca2+-PPP3/calcineurin-TFEB axis
Source: Autophagy. 2021 Aug 2;18(4):799–815. doi: 10.1080/15548627.2021.1956123 (PMC9037459; doi:10.1080/15548627.2021.1956123)
Supplement: Supplemental Material [file KAUP_A_1956123_SM4892.zip › Supplementary information/Zummo supplemental legends and table - R7.docx]

**Figure S1.** Impact of siRNA knockdown of *Rapgef4* on cAMP in INS-1E. (**A**) INS-1E were cultured with or without GLT for 16 h prior to the addition of 100 nM Ex4 for 30 min. cAMP was determined via ELISA and is expressed as pmol/mg protein. (**B**) INS-1E were treated with scrambled siRNA (siScr) or siRNA against *Rapgef4* (si*Rapgef4*) for 48 h prior to incubation with 100 nM Ex4 for 30 min. cAMP was determined via ELISA and is expressed as pmol/mg protein. All data are mean ± SEM of four individual experiments. Statistical analysis was performed using a two-way ANOVA followed by Bonferroni's post-hoc test. # P < 0.05, ### P < 0.005 effect of Ex4.

**Figure S2.** Expression of *RAPGEF3* and *RAPGEF4* in mouse and human pancreatic beta-cells. (**A**) The publicly available transcriptomic database Tabula Muris (<https://tabula-muris.ds.czbiohub.org/>) (ref PMID 30283141) [32] was used to check the expression of *Rapgef3* and *Rapgef4* in β-cells and other murine pancreatic cell populations. *Ins1* specific expression in β-cells (“Type B cells”) was used as control. Image credit: Tabula Muris. (**B**) Expression levels of *RAPGEF3* and *RAPGEF4* in human pancreatic cell populations were verified using the public available transcriptomic database from the Human Protein Atlas (<https://www.proteinatlas.org/>) (ref PubMed: 25613900) [33]. Pancreatic cell populations are identified by the expression of specific genes: β-cells were grouped with the other endocrine cells (c-6, in bold) and this cluster defined by the expression of *INS* (insulin), *GCG* (glucagon), *PPY* (pancreatic polypeptide) or *CHGA* or *CHGB* (chromogranin A or B). Image modified from: Human Protein Atlas (version 20.1) (https://www.proteinatlas.org/ENSG00000091428-RAPGEF4/celltype/pancreas).

**Figure S3.** Representative images of total cellular Ca^2+^ imaging. (**A-C**) INS-1E were pre-incubated with 2 μM Fluo-8 AM for 35 min followed by 20 min washout with Krebs-Hepes. Basal readouts were taken at Time 0 before cells were stimulated with either 15 mM glucose (**A**), 15 mM glucose + 100 nM Ex-4 (**B**) or with 0.5 μM Ionomycin (**C**). Changes in fluorescence were detected by fluorescence microscopy and expressed as ΔF/F_0_. Images are representative of four individual experiments at Basal (0 time) and Bl, Ex4 or Iono (100 s). Output is converted to a false-color Fire-LUT scale. White arrowheads denote responding cells. Scale bars: 50 µm.

**Figure S4.** Representative images of lysosomal Ca^2+^ imaging. INS-1E were transfected with GCaMP3-ML1 (GCaMP) for 24 h prior to addition of Rhod-2-AM (Rhod2) for 30 min followed by 30 min washout in Tyrode’s solution at 11 mM glucose. Cells were then stimulated with 100 nM Ex4 (**A**) or media only (Bl) (**B**) for 90 s. Specificity of the construct was confirmed by addition of 400 μM glycyl-L-phenylalanine-beta-naphthylamide (GPN) followed by 66 mM CaCl_2_ (Ca^2+^). Changes in fluorescence at Ex:490, Em:525 (for GCaMP) and Ex:550, Em:580 (for Rhod-2-AM) were detected by confocal microscopy (Nikon A1R) and expressed as ΔF/F_0_. Images are representative of five individual experiments at Basal (0 time), Bl or Ex4 (67 s), GPN (129 s) and Ca^2+^ (156 s). Output is converted to a false-color Fire-LUT scale. White arrowheads denote responding cells. Scale bars: 10 µm.

**Figure S5.** Exendin-4 stimulates release of endoplasmic reticulum Ca^2+^. INS-1E were transfected with ER-GCaMP6-150 (GCaMP) and treated for 24 h prior to addition of Rhod-2-AM (Rhod2) for 30 min followed by 30 min washout in Tyrode’s solution at 11 mM glucose. Cells were then stimulated with 100 nM Ex4 (**A and C**) or media only (Bl) (**B and D**) for 90 s. Specificity of the construct was confirmed by addition of 10 μM ionomycin (Iono) followed by 66 mM CaCl_2_ (Ca^2+^). Changes in fluorescence at Ex:490, Em:525 (for GCaMP) and Ex:550, Em:580 (for Rhod-2-AM) were detected by confocal microscopy (Nikon A1R) and expressed as ΔF/F_0_. Images are representative of five individual experiments at Basal (0 time), Bl or Ex4 (60 s), Iono (124 s) and Ca^2+^ (153 s). Output is converted to a false-color Fire-LUT scale. White arrowheads denote responding cells. Scale bars: 10 µm.

**Figure S6.** Exendin-4 stimulates uptake of Ca^2+^ across the plasma membrane. INS-1E were transfected with pGP-CMV-GCaMP6s (GCaMP) for 24 h prior to addition of Rhod-2-AM (Rhod2) for 30 min followed by 30 min washout in Tyrode’s solution at 11 mM glucose. Cells were then stimulated with 100 nM Ex4 (**A and C**) or media only (Bl) (**B and D**) for 90 s. Specificity of the construct was confirmed by addition of 1 μM Bay K8644 (Bay) followed by 66 mM CaCl_2_ (Ca^2+^). Changes in fluorescence at Ex:490, Em:525 (for GCaMP) and Ex:550, Em:580 (for Rhod-2-AM) were detected by confocal microscopy (Nikon A1R) and expressed as ΔF/F_0_. Images are representative of five individual experiments at Basal (0 time), Bl or Ex4 (57 s), Bay (123 s) and Ca^2+^ (153 s). Output is converted to a false-color Fire-LUT scale. White arrowheads denote responding cells. Scale bars: 10 µm.

**Figure S7.** Representative images of pancreas tissue from *in vivo* study. C57BL/KsJ *db/db* mice were injected twice daily with either saline or exendin-4(1–39) at 25 nmol/kg bw for 21 d [49]. Aged matched lean control mice (C57BL/6J) treated with saline were used as non-diabetic controls. Pancreatic tissue was stained for: (**A**) DAPI (blue), SQSTM1 (red), INS (magenta); (**B**) DAPI (blue), TFEB (green), INS (red); (**C**) DAPI (blue), CTSD (red), INS (magenta), and imaged using confocal microscopy. Images are representative of 10-15 islets imaged per condition. Scale bars: 20 µm.

**Table S1.** Inhibitor and Activator details.

| **Target** | **Chemical name** | **Cat. number** | **Company** |
| --- | --- | --- | --- |
| **AKT1** | AKT inhibitor VIII  Isozyme Akti-1/2 | 124018 | Sigma Aldrich |
| **AMPK** | Compound C | BML-EI369-0005 | Enzo Life Sciences |
| **Ca^2+^** | BAPTA | BML-CA411-0025 | Enzo Life Sciences |
| **Ca^2+^** | Bay K 8644 | 1544 | Biotechne Europe Ltd |
| **Ca^2+^** | Gly-Phe-naphthylamide | SC-252858 | Santa Cruz Biotechnology |
| **Ca^2+^** | Ionomycin | I3909 | Sigma Aldrich |
| **PPP3** | FK506 | ALX-380-008-M001 | Enzo Life Sciences |
| **CAMKK** | STO-609 | SC-202820 | Santa Cruz Biotechnology |
| **RAPGEF3-RAPGEF4** | ESI-09 | SML0814 | Sigma Aldrich |
| **RAPGEF4** | ESI-05 | SML1907 | Sigma Aldrich |
| **RAPGEF3-RAPGEF4** | 8-CPT-2Me-cAMP | 1645/1 | R&D Systems |
| **RAPGEF4** | Sp-8-BnT-cAMPS | B 046-05 | Biolog Life Science Institute |
| **RAPGEF4** | Sp-8-BnT-2’-O-Me-cAMPS | B 056-01 | Biolog Life Science Institute |
| **PRKA/PKA** | Rp-cAMPs | SC-24010 | Santa Cruz Biotechnology |
|  |  |  |  |

**Table S2.** Patient details.

| **Identifier** | **Isolation Centre** | **Sex** | **Age** | **BMI** | **Experiment** |
| --- | --- | --- | --- | --- | --- |
| **LDIS177** | Edmonton | F | 36 | 28.8 | Fig1H |
| **LDIS178** | Edmonton | M | 61 | 29 | Fig1H |
| **LDIS182** | Edmonton | F | 73 | 27.4 | Fig1H |
| **LDIS256** | Newcastle | M | 53 | 25 | Fig5C, Fig7H-J |
| **R286** | Edmonton | M | 22 | 22.4 | Fig5C, Fig7H-J |
| **LDIS266** | Newcastle | F | 65 | 28.15 | Fig5C |
| **LDIS268** | Newcastle | M | 37 | 20.52 | Fig7H-J |
| **LDIS274** | Edinburgh | F | 47 | 37.22 | Fig7H-J |
| **LDIS292** | Oxford | F | 42 | 37.00 | Fig5C, Fig7H-J |

**Table S3.** Primer sequences for real time RT-PCR.

| **Gene** | **FWD Primer** | **REV Primer** |
| --- | --- | --- |
| ***Rap1a*** | GTG CTT TTC CTT TCC TTC CC | CTT GCC GTC TGC TTT TTT C |
| ***Rap1b*** | AGA TTC TCA CTG TGC CTC C | ATT ACT TCC CAC ATT CAC |
| ***Ppia/ Cyclophilin a*** | ATG GCA CTG GTG GCA AGT CC | TTG CCA TTC CTG GAC CCA AA |
