# Supplementary figures and images for: Exendin-4 stimulates autophagy in pancreatic β-cells via the RAPGEF/EPAC-Ca2+-PPP3/calcineurin-TFEB axis
Source: Autophagy. 2021 Aug 2;18(4):799–815. doi: 10.1080/15548627.2021.1956123 (PMC9037459; doi:10.1080/15548627.2021.1956123)

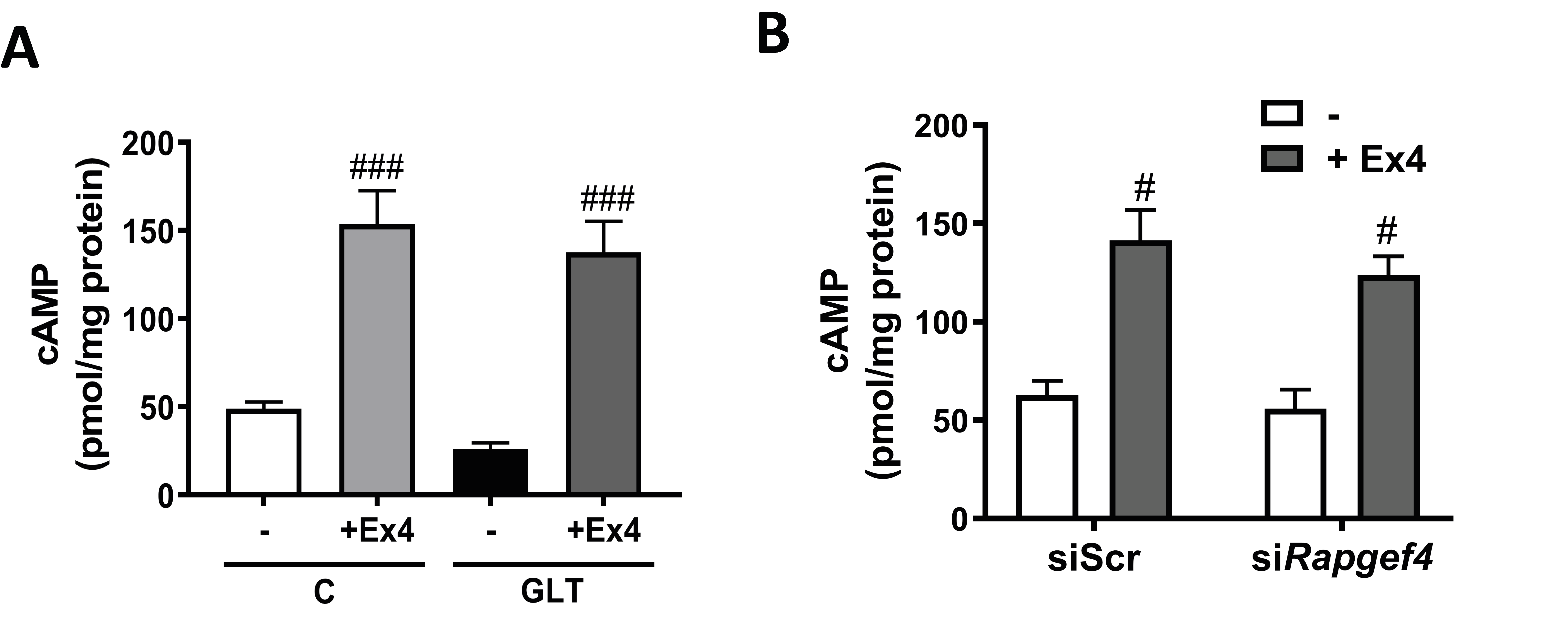

Supplement: Supplemental Material [file KAUP_A_1956123_SM4892.zip › Supplementary information/supplementary figure 1.tif]

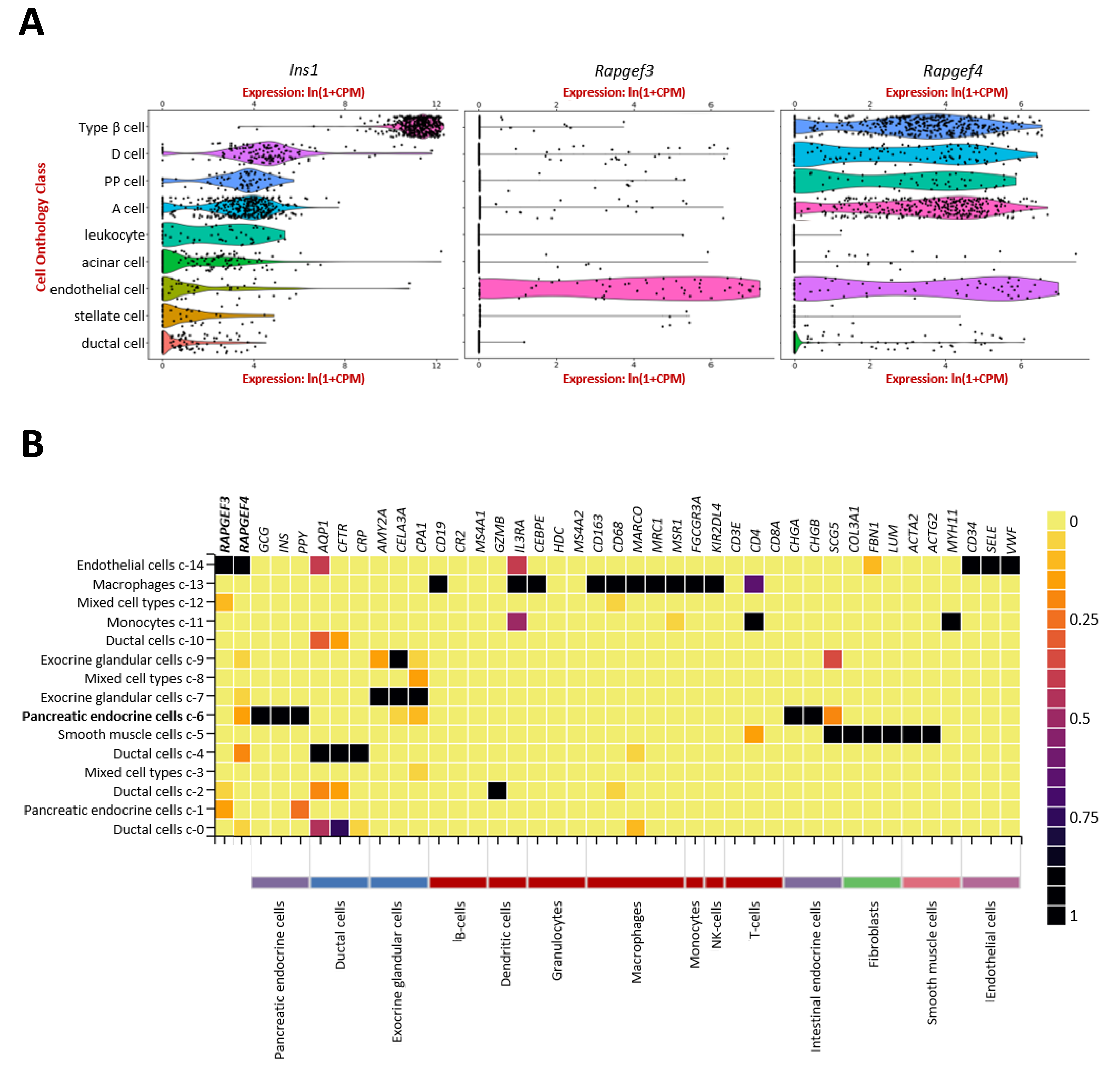

Supplement: Supplemental Material [file KAUP_A_1956123_SM4892.zip › Supplementary information/supplementary figure 2.tif]

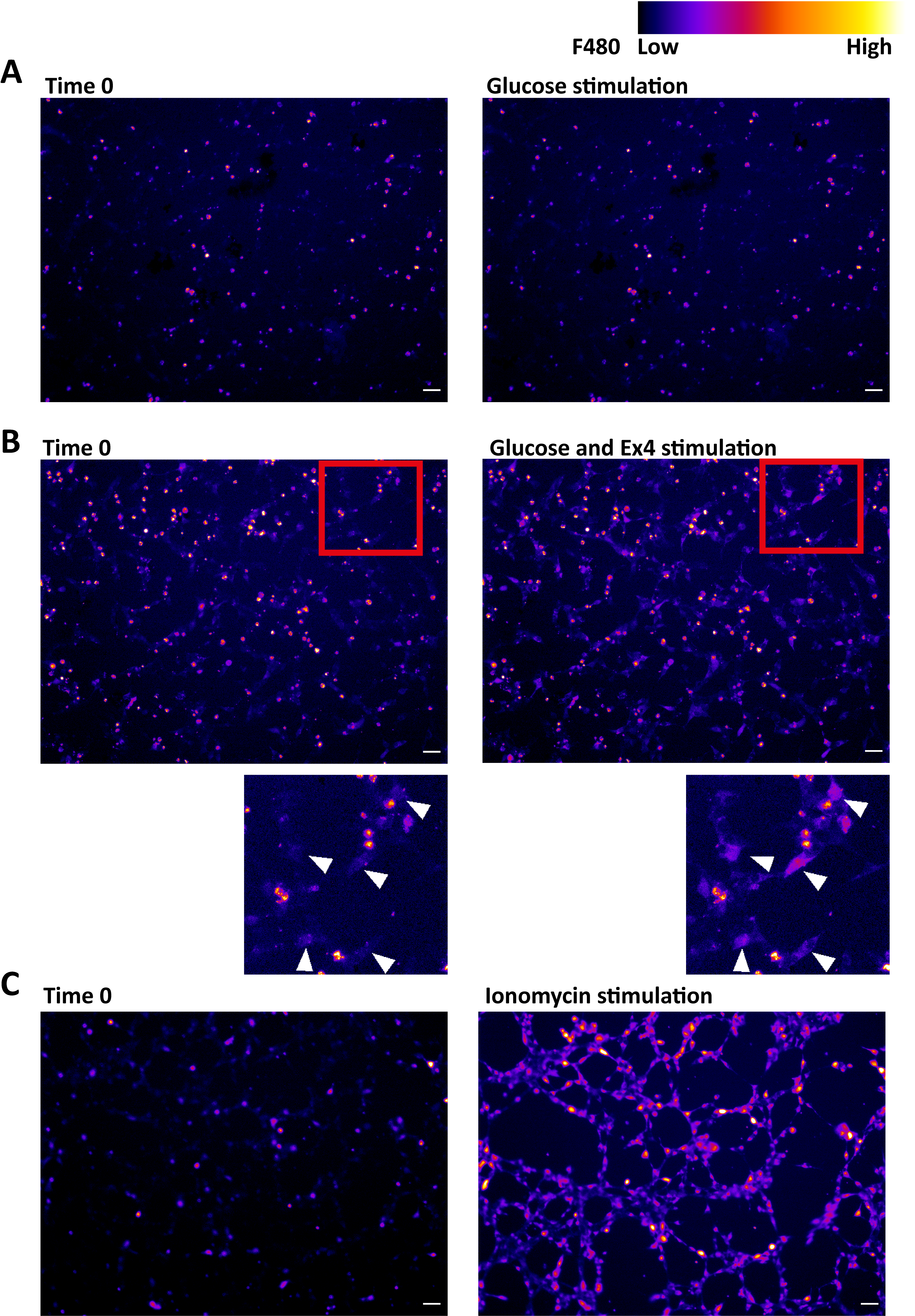

Supplement: Supplemental Material [file KAUP_A_1956123_SM4892.zip › Supplementary information/supplementary figure 3.tif]

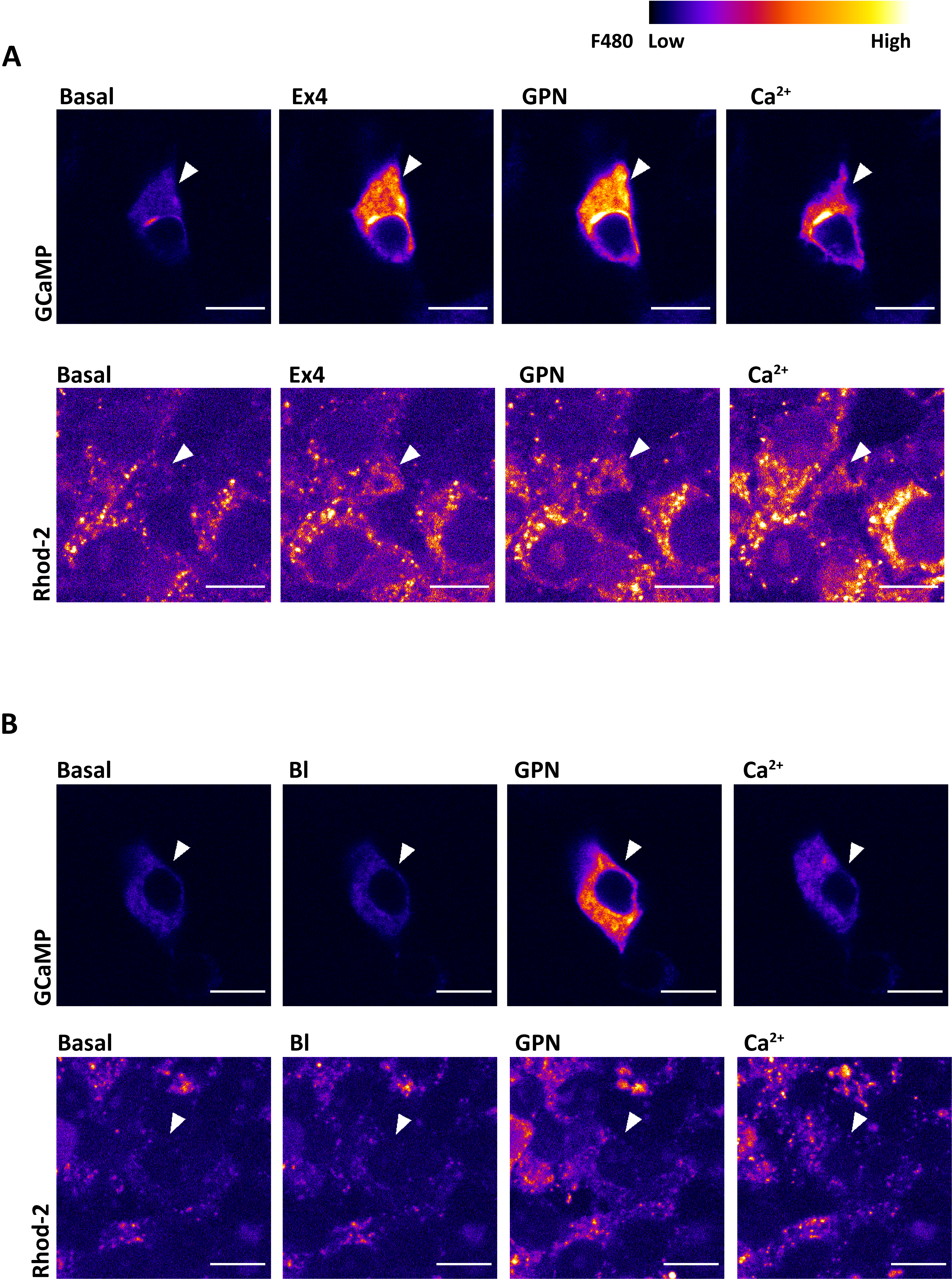

Supplement: Supplemental Material [file KAUP_A_1956123_SM4892.zip › Supplementary information/supplementary figure 4.tif]

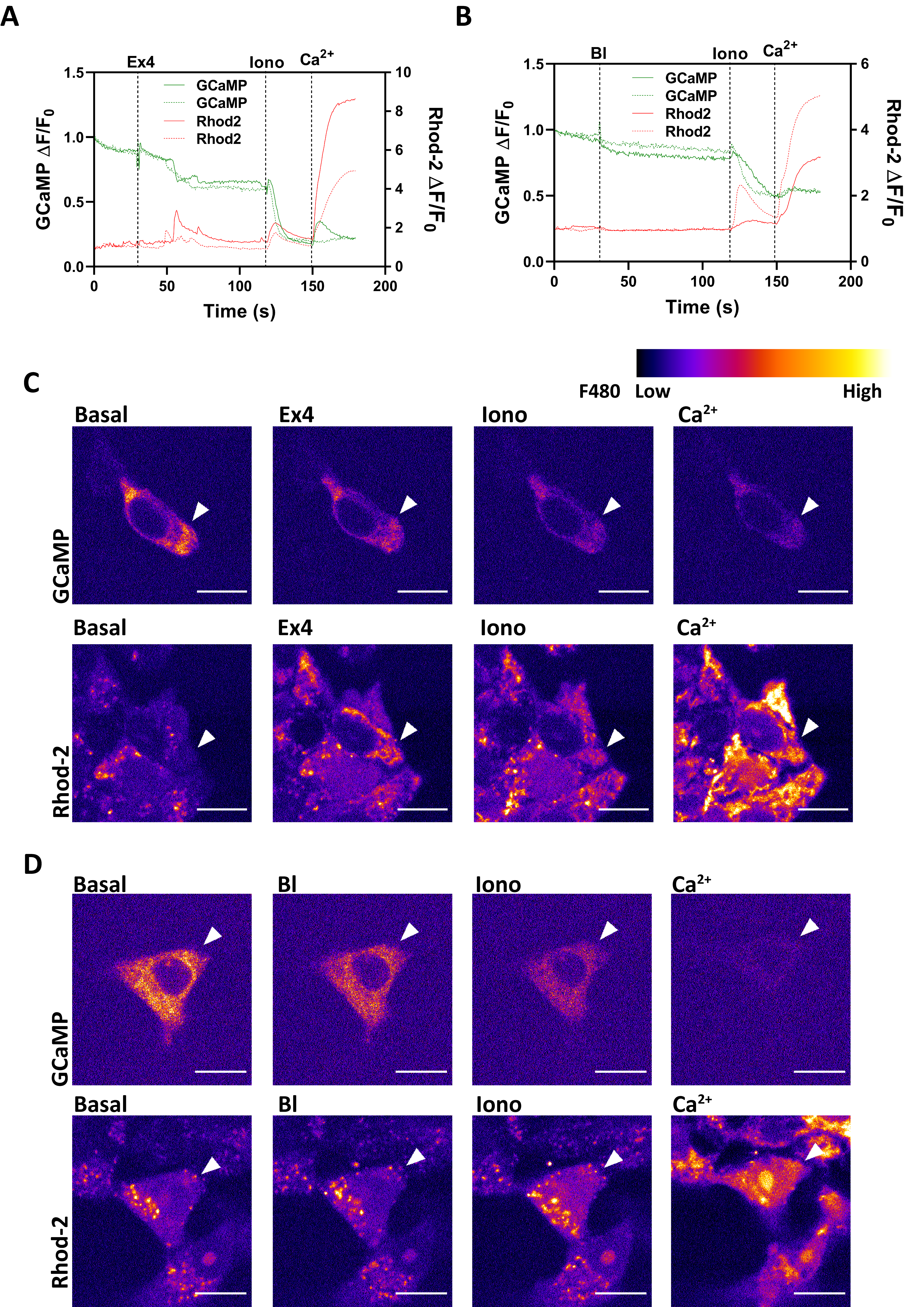

Supplement: Supplemental Material [file KAUP_A_1956123_SM4892.zip › Supplementary information/supplementary figure 5.tif]

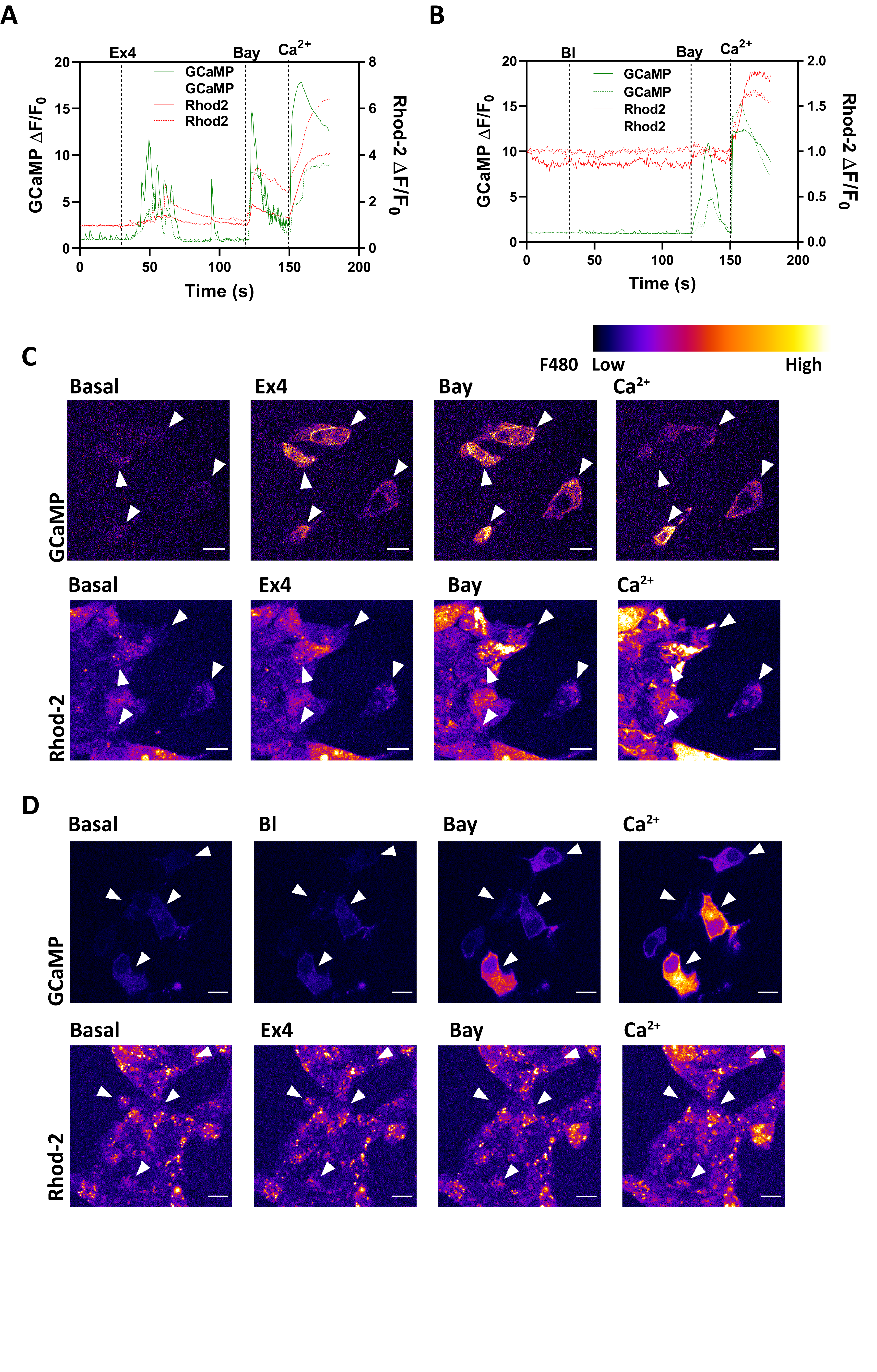

Supplement: Supplemental Material [file KAUP_A_1956123_SM4892.zip › Supplementary information/supplementary figure 6.tif]

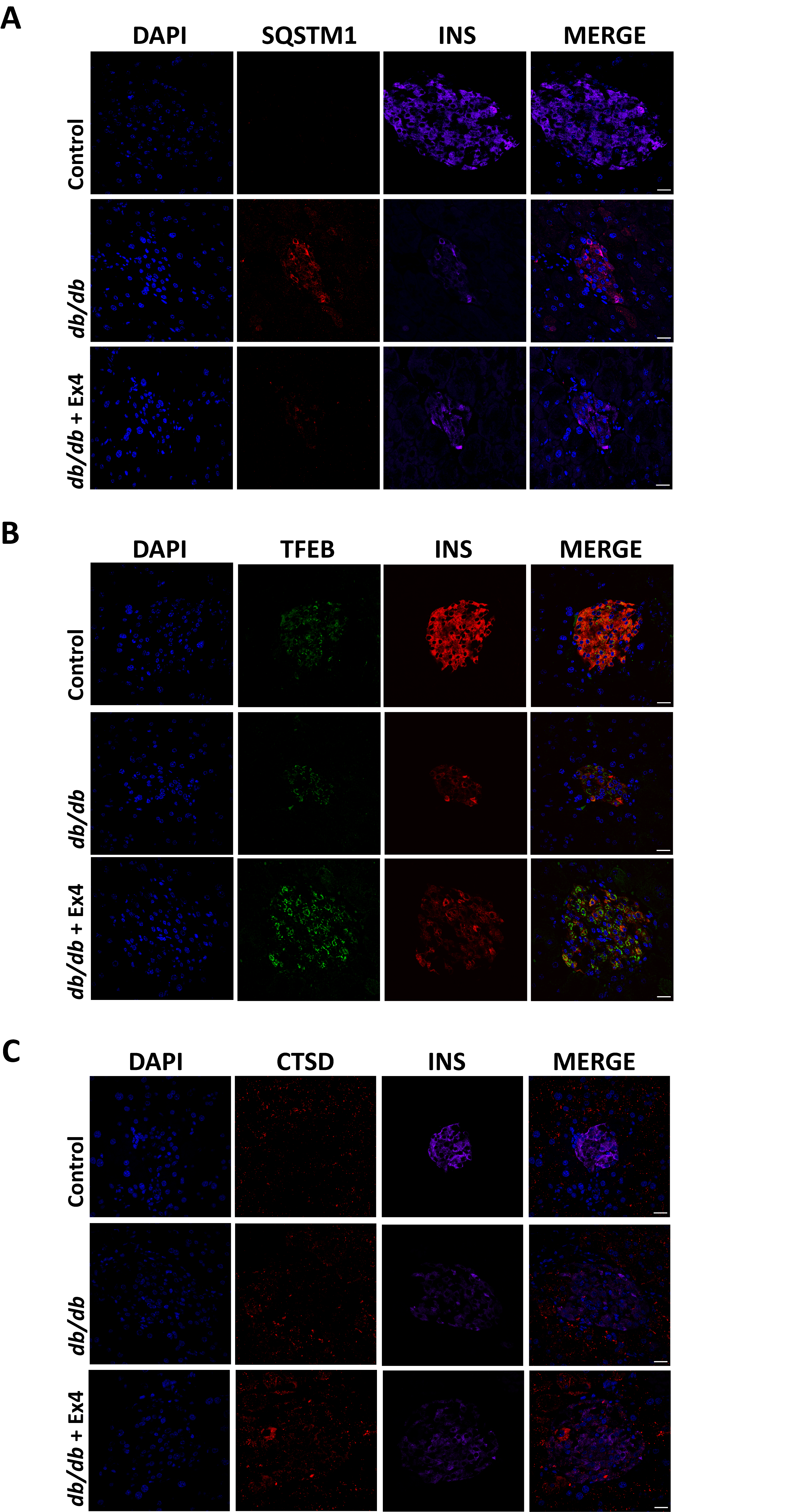

Supplement: Supplemental Material [file KAUP_A_1956123_SM4892.zip › Supplementary information/supplementary figure 7.tif]
